# Modeling geogenic and atmospheric nitrogen through the East River Watershed, Colorado Rocky Mountains

**DOI:** 10.1371/journal.pone.0247907

**Published:** 2021-03-24

**Authors:** Taylor Maavara, Erica R. Siirila-Woodburn, Fadji Maina, Reed M. Maxwell, James E. Sample, K. Dana Chadwick, Rosemary Carroll, Michelle E. Newcomer, Wenming Dong, Kenneth H. Williams, Carl I. Steefel, Nicholas J. Bouskill

**Affiliations:** 1 Earth and Environmental Sciences Area, Lawrence Berkeley National Laboratory, Berkeley, CA, United States of America; 2 School of the Environment, Yale University, New Haven, CT, United States of America; 3 Civil and Environmental Engineering, Princeton Environmental Institute, Princeton University, Princeton, NJ, United States of America; 4 Norwegian Institute for Water Research (NIVA), Grimstad, Norway; 5 Department of Earth System Science, Stanford University, Stanford, CA, United States of America; 6 Desert Research Institute, Reno, NV, United States of America; 7 Rocky Mountain Biological Laboratory, Crested Butte, CO, United States of America; Durham University, UNITED KINGDOM

## Abstract

There is a growing understanding of the role that bedrock weathering can play as a source of nitrogen (N) to soils, groundwater and river systems. The significance is particularly apparent in mountainous environments where weathering fluxes can be large. However, our understanding of the relative contributions of rock-derived, or geogenic, N to the total N supply of mountainous watersheds remains poorly understood. In this study, we develop the High-Altitude Nitrogen Suite of Models (HAN-SoMo), a watershed-scale ensemble of process-based models to quantify the relative sources, transformations, and sinks of geogenic and atmospheric N through a mountain watershed. Our study is based in the East River Watershed (ERW) in the Upper Colorado River Basin. The East River is a near-pristine headwater watershed underlain primarily by an N-rich Mancos Shale bedrock, enabling the timing and magnitude of geogenic and atmospheric contributions to watershed scale dissolved N-exports to be quantified. Several calibration scenarios were developed to explore equifinality using >1600 N concentration measurements from streams, groundwater, and vadose zone samples collected over the course of four years across the watershed. When accounting for recycling of N through plant litter turnover, rock weathering accounts for approximately 12% of the annual dissolved N sources to the watershed in the most probable calibration scenario (0–31% in other scenarios), and 21% (0–44% in other scenarios) when considering only “new” N sources (i.e. geogenic and atmospheric). On an annual scale, instream dissolved N elimination, plant turnover (including cattle grazing) and atmospheric deposition are the most important controls on N cycling.

## 1. Introduction

There is a growing understanding of the importance of bedrock weathering as a source of nitrogen (N) to groundwater and river systems, particularly in mountain environments where weathering fluxes can be large [1–4]. Houlton et al. [5] recently estimated that 92–110 Pg N are stored in the top 1m of rock worldwide, and that 19–31 Tg N yr^-1^ are mobilized (i.e. made available via weathering) from near-surface rocks annually, nearly tripling previous estimates of global rock-derived N fluxes. In N-limited mountainous watersheds, bedrock-derived N may represent a majority of N available for plant and microbial use [6, 7], particularly given rock-derived N can be weathered to a bioavailable form such as dissolved organic N (DON) or ammonium (NH_4_^+^), which is readily utilized by plants and microbes [4, 7]. However, our understanding of the relative contributions of geogenic N to the total N supply of mountainous watersheds and its contribution to dissolved N loads supplied downstream remains unclear.

One of the main limitations restricting our ability to quantify the fate and transport of mountain N is an absence of watershed-scale biogeochemical models that directly focus on high altitude regions, specifically incorporating hydrological, geological, biogeochemical, and climatic drivers relevant to mountain environments. The majority (>80%) of watershed N models have been constructed for application to agricultural systems [8], where riverine N loads can usually be predicted based on fertilizer application regimes [9, 10]. In near-pristine mountain environments where N concentrations are considerably lower, and often limiting to ecosystem productivity [11], N mass balance is driven by a complex series of interacting drivers, including bedrock weathering [5, 6], plant uptake, including direct organic N uptake [12, 13], plant storage and release [14], the timing of spring snowmelt [15], changes to atmospheric deposition [16], denitrification [17, 18], and erosion of particulate N off steep hillslopes and mountainsides [19, 20]. Though there are models (e.g. INCA) that have been selectively adapted to predict N fluxes in mountain watersheds [21, 22], our ability to predict spatiotemporally dynamic changes to mountain N concentrations lags behind agricultural systems, particularly in systems with potentially large sources of geogenic N, where information on mineralogy and weathering rates are required.

Calibrating models using ‘end-of-pipe’ stream nutrient measurements results in the possibility of equifinality, i.e. the occurrence of multiple parameter combinations that predict the same stream nutrient concentrations over time. At present, there is no satisfactory solution for both identifying and quantifying all possible calibration parameter combinations, due in large part to the inability to constrain reaction and transport rates representative of entire sub-watersheds. Auto-calibration approaches such as Markov Chain Monte Carlo (MCMC) have been proposed to identify equifinality [23–25], but are difficult to apply due to the model complexity, the high spatiotemporal frequency of measurements needed, and the fact that auto-calibration may actually result in less realistic predictions of nutrient dynamics than manual calibration [26–28]. Nevertheless, it is important for complex watershed nutrient models to recognize and identify the possibility of equifinality in order to minimize model uncertainty, and we can use manual calibrations to explore key regions of the parameter space.

In this study, we focus on the East River Watershed (ERW) north of Crested Butte in the Colorado Rocky Mountains. The ERW is a relatively pristine headwater tributary to the Gunnison River in the Upper Colorado River basin, which provides 10% of the flow to the Gunnison River, which, in turn, provides 40% of the flow to the Colorado River at the Colorado-Utah border [29]. The ERW is predominantly underlain by a Cretaceous age, N-rich Mancos shale bedrock, which offers a unique opportunity to quantify the fate and transport of both geogenic and atmospheric N at the watershed scale. Mancos shale weathering in the ERW occurs primarily due to abiotic processes controlled by the water table depth [30, 31]. Leaching experiments of Mancos cores from the ERW indicate that shale-N is primarily organic N and ammonium [30]. Seasonal saturation of the shale, following the snowmelt-induced water table rise, results in the dissolution of organic N and the desorption of NH_4_^+^ from clay minerals (primarily illite and smectite [32]. Following mobilization, experimental evidence indicates that DON and NH_4_^+^ are both readily mineralized and nitrified by microflora, driving rapid NO_3_^-^ production [30].

Here we develop the High-Altitude Nitrogen Suite of Models (HAN-SoMo), a watershed-scale, process-based N ensemble of box models representing the hydrologic and dissolved N cycles, which discretizes N cycling into sub-watersheds (i.e., is semi-distributed). Given available measurements, we focus specifically on dissolved N species, but due to the importance of particulate N in mountain environments, devote a portion of the discussion to hypotheses related to particulate N loads in stream. This is the first study to utilize all available ERW dissolved N species time series data for both surface and subsurface waters to quantify whole watershed N cycling. Transient hydrological input parameters are constrained via coupling to the three-dimensional groundwater-surface water model ParFlow [33], which is further coupled to the Community Land Model (CLM) [34] that has been specifically developed at high resolution for the ERW [35]. Herein, we quantify the relative contributions of atmospheric deposition and bedrock weathering to dissolved N exports downstream. We further quantify storage, loss, and N species transformation fluxes including plant uptake and release via litter decomposition, denitrification, nitrification, and mineralization. In addition to the coupling of these models, we also employ the high-resolution spatiotemporal monitoring scheme in place at the ERW since 2014 to calibrate the model. This includes hourly measurements of discharge and daily-to-monthly stream water N measurements at tributary confluences and three main reaches on the East River, and groundwater N measurements from several locations. The goal of our study is to determine the relative contribution of different N fluxes, including the magnitude and fate of N derived from shale weathering, to total N-export. from a pristine mountainous watershed.

## 2. Methods

### 2.1 Study watershed

**Fig 1:**
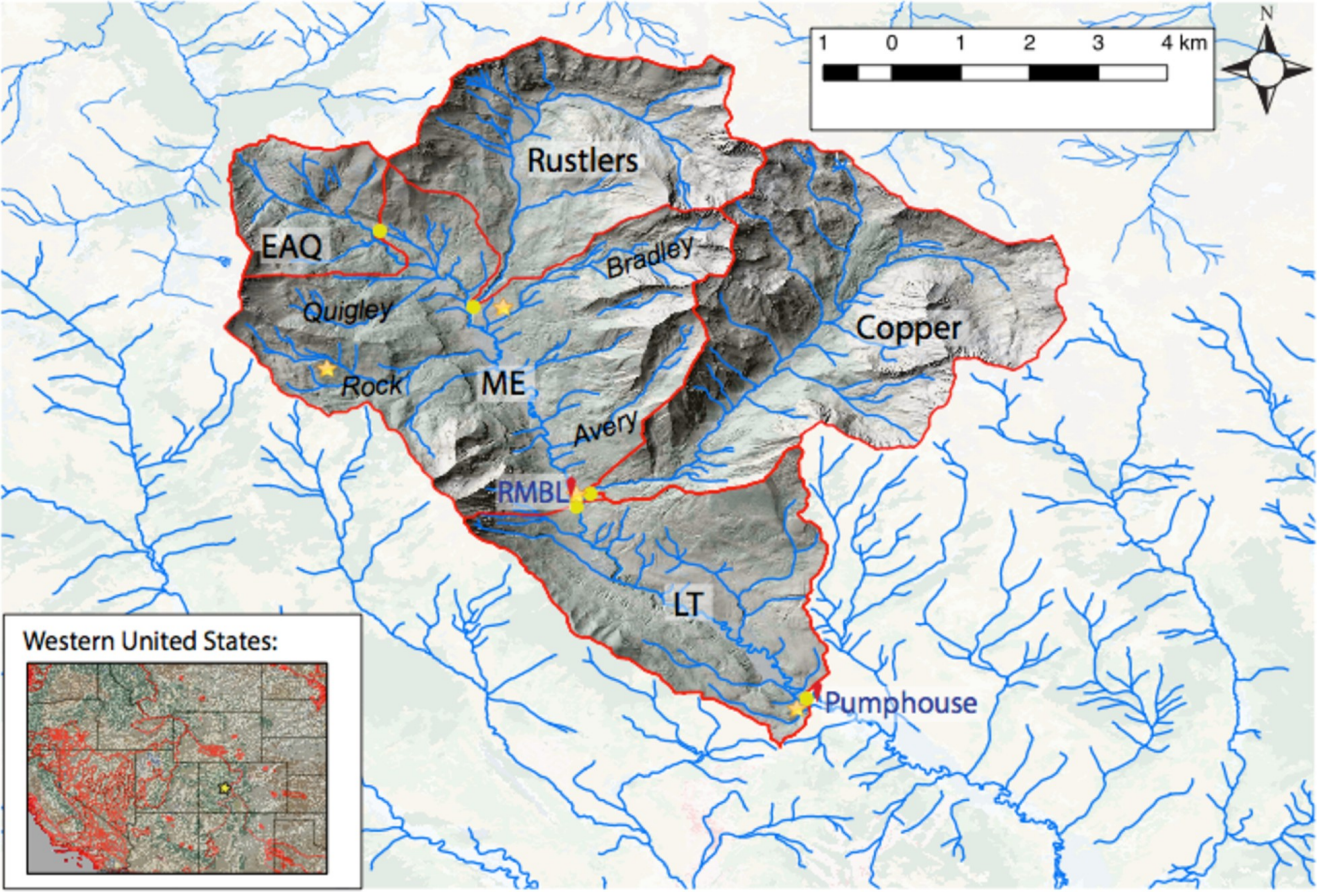
Map of East River Watershed, Colorado, with all sampling locations (yellow circles) and sub-watersheds discretized in HAN-SoMo (red boundaries). Orange stars indicate piezometer locations, and red markers show the Rocky Mountain Biologic Lab (RMBL) in Gothic, and the Pumphouse (PH). Italicized names are streams and normal case are sub-watersheds. Inset: Red line show major watershed boundaries, black lines show state boundaries, and yellow star indicates East River Watershed location.

The ERW is an intensely monitored observational watershed, and has been heavily instrumented [36], with over 1600 stream water dissolved N concentration measurements in five key sub-catchments, spanning a time period 2014–2020. This study is focused on an 85 km^2^ region of the watershed around the Rocky Mountain Biological Laboratory (RMBL) and specifically the encompassing watershed area located north of a pumphouse (PH), used to extract water for use by the adjacent municipality of Mt. Crested Butte, Colorado (Fig 1). This region includes alpine, sub-alpine, and montane ecosystems ranging in elevation from 2760m to 4120m. Eight perennial tributaries drain into the East River (ER) upstream of PH: Rustlers, Copper, Gothic, Quigley, Rock, Marmot, Bradley, and Avery Creeks. The watershed receives 670–1200 mm of precipitation annually, depending on the monitoring location within the watershed, with about 70% as snow [37], and the majority of the remaining 30% during monsoonal rains in late summer and early fall. Land cover ranges from barren rock to quaking aspen (*Populus tremuloides*) stands, Engelman spruce (*Picea engelmannii*) and subalpine fir (*Abies lasiocarpa*) mixed forest, dry shrub/scrub, and meadows away from stream networks, and riparian areas predominately characterized by woody shrub vegetation, dominated by members of the *Salix* genus (willows), with interspersed herbaceous wetlands (Table 1). N-fixing plants, including members of the lupine genus (*Lupinus argenteus*, *Lupinus bakeri*), sweet pea (*Lathyrus latifolius*), and American vetch (*Vicia americana*), are unevenly distributed throughout the watershed’s meadows.

**Table 1:** Sub-watershed properties derived from the USGS National Land Cover Database. Mancos Shale (%) refers to the proportion of the sub-watershed that is underlain by Mancos Shale bedrock or saprolite within the top 8m, as estimated by Carroll et al. [37].

Parameter	EAQ	Rustlers	Copper	ME	LT
Surface area (m^2^)	5.27 x 10^6^	1.48 x 10^7^	2.37 x 10^7^	2.61 x 10^7^	1.49 x 10^7^
Deciduous cover (%)	13.1	1.3	2.3	12.5	33.0
Coniferous cover (%)	20.5	18.9	19.4	35.3	13.5
Meadow cover (%)	31.0	46.8	27.8	27.4	42.2
Willowy wetland cover (%)	12.2	11.8	4.9	5.8	7.5
Mancos Shale (%)	70	8	1	18	18

The ERW is mainly underlain by Mancos shale [6], which outcrops throughout the watershed and has high N concentrations (solid concentrations ~1150–1400 mg N kg^-1^). The watershed vadose zone largely developed atop of a region of weathered Mancos shale-derived saprolite, with additional area covered by a mixture of colluvium and glacial deposits. Less prominent bedrock formations include sandstone, conglomerate, and Oligocene quartz monzonites and granodiorites, including two large laccoliths that form the mountains on the southwestern edge of the watershed. Finally, a herd of cattle numbering ca. 500 head roam the watershed from late July to early October, grazing between PH and RMBL for approximately one month before moving upstream of RMBL and into tributary sub-watersheds for the remainder of the grazing period (J. Reithel, personal communication).

### 2.2 Data collection and analyses

All research activities and infrastructure used to support the findings presented here were performed under Special Use Permit GUN1132 issued by the United States Forest Service to Lawrence Berkeley National Laboratory. High-frequency (every 1–3 days) stream water samples were collected at PH from October 2014 to October 2018 and analyzed for nitrate (NO_3_^-^) concentration. During the same time interval, NO_3_^-^ analysis was performed on samples collected at weekly to monthly intervals at the confluences of each of the other tributaries with the ER main stem, as well as on the ER above the confluence with Quigley Creek (EAQ), and on the ER at RMBL. Stream water recovered from each site were filtered (0.45 *μ*M) and analyzed for NO_3_^-^ concentrations via anion chromatography (Dionex, Corp. ICS-2100, Sunnyvale, CA) using an AS-18 anion exclusion column; the method detection limit based on calibration standards for nitrate is 0.1μM. Stream water samples for analysis of total dissolved nitrogen (TDN) and ammonium (NH_4_^+^) were collected at the same locations less frequently (weekly to monthly) and filtered (0.45 *μ*M). Stream discharge was measured at hourly intervals at LT, Copper, EAQ, and Rustlers according to the methods described in Carroll et al. [37] (note that long-term discharge monitoring was not possible at ME). Groundwater samples were collected intermittently throughout the watershed using installed piezometers at PH, RMBL, Bradley and Rock Creeks (Fig 1). TDN was analyzed via chemiluminescence using a Shimadzu Total Nitrogen Module combined with a TOC-VCSH analyser (Shimadzu Corporation, Japan). NH_4_^+^ was measured colorimetrically using a Lachat’s QuikChem 8500 Series 2 Flow Injection Analysis System (LACHAT Instruments, QuickChem 8500 series 2, Automated Ion Analyzer, Loveland, Colorado). Dissolved organic nitrogen (DON) was calculated by subtracting NH_4_^+^ and NO_3_^-^ from TDN.

### 2. 3 Model overview

The suite of models consists principally of box models representing the hydrology and N dynamics in the river, soil water (vadose zone) and groundwater for each subwatershed *i*. The ERW is discretized into five sub-watersheds (Fig 1): the largest tributaries Copper Creek and Rustlers Gulch, and the main East River headwaters above the confluence with Quigley Creek (EAQ), the Middle East (ME) River catchment from EAQ to RMBL below its confluence with Copper Creek, including the minor tributaries Avery, Marmot, Bradley, Rock, Quigley and Gothic Creeks, and the lower triangle-shaped (LT) sub-watershed downstream of RMBL to PH. Hydrological parameters are constrained using outputs fed from the ParFlow model, coupled to the Community Land Model (ParFlow-CLM). Both ParFlow-CLM and the N models are solved with hourly timesteps from Oct. 1, 2014, run for 4 years (1462 days), and the numerical N model is solved using Runge-Kutta 4 integration.

### 2.4 Model structure

#### 2.4.1. ParFlow-CLM

ParFlow is a three-dimensional integrated hydrological model that simulates subsurface and surface water flows by solving the Richards’ equation and shallow surface water equations [38–41]. ParFlow contains a coupled land surface module, CLM, which solves the energy balance for many land surface processes. Canopy water balance, losses and additions from evapotranspiration (ET), precipitation and snowmelt are communicated with ParFlow at every timestep [33, 34, 42–44]. ParFlow-CLM has been applied to the ERW at 1km and 100m resolution; full details of the model construction and performance can be found in Foster and Maxwell [35] and Foster et al. [45]. Here, we use both input and output from the 100m resolution model run. The subsurface is discretized into five layers across the entire watershed, the top three are soil layers of depths 0.1m, 0.3m and 0.6m, while the bottom two are geological layers of 8m and 21m, with the deepest 21m representing fractured bedrock.

Pressure head output from ParFlow-CLM was spatially integrated for each sub-watershed *i* (Fig 1) to determine the total volume of water stored in the groundwater, *V*_*g*,*i*_, vadose zone, *V*_*s*,*i*_, and surface water, *V*_*r*,*i*_, at hourly intervals. Movement into and out of the vadose zone is quantified with two fluxes from ParFlow-CLM, also at hourly intervals: infiltration into the vadose zone, *Infilt*_i_, and evapotranspiration from the soil layers, *ET*_*s*,*i*_. Infiltration includes snowmelt water, precipitation, and runoff from adjacent cells that enters the vadose zone, while exfiltration (i.e. negative infiltration) includes vadose zone water that exits to the surface via saturation excess. Surface water pressure head values were converted to discharge (flows) using Manning’s equation for the outlet of each sub-watershed. Discharge is a function of the representative slope values of each outlet, and the Manning *n* value of that cell, as parameterized by land cover type. As discussed in Foster and Maxwell [35], Manning *n* and hydraulic conductivity were used constrain system-wide discharge by manual calibration with stream-level observations to control the dynamics of the streams.

Soil and air temperature (T_soil_ and T_air_, °C) were output as hourly spatial averages for each sub-watershed. Air temperatures are derived from PRISM datasets [46], interpolated to hourly resolution using phase two of North American Land Data Assimilation (NLDAS-2) forcing [47], which are available at 1/8^th^-degree at hourly time steps, and were interpolated and downscaled to match the discretization of the ParFlow-CLM model. Soil temperature is solved in CLM using the heat diffusion equation and a subsurface heat flux with the Fourier law for heat conduction over the top 2 meter of the model for both soil and snow layers [48]. Stream water temperatures were calculated using the empirical relationship given in Lauerwald et al. [49]. We calculate a single groundwater temperature (T_gw,_ °C) for the entire ERW by averaging sub-watershed soil temperatures at each timestep.

#### 2.4.2. Aggregated hydrology

**Fig 2:**
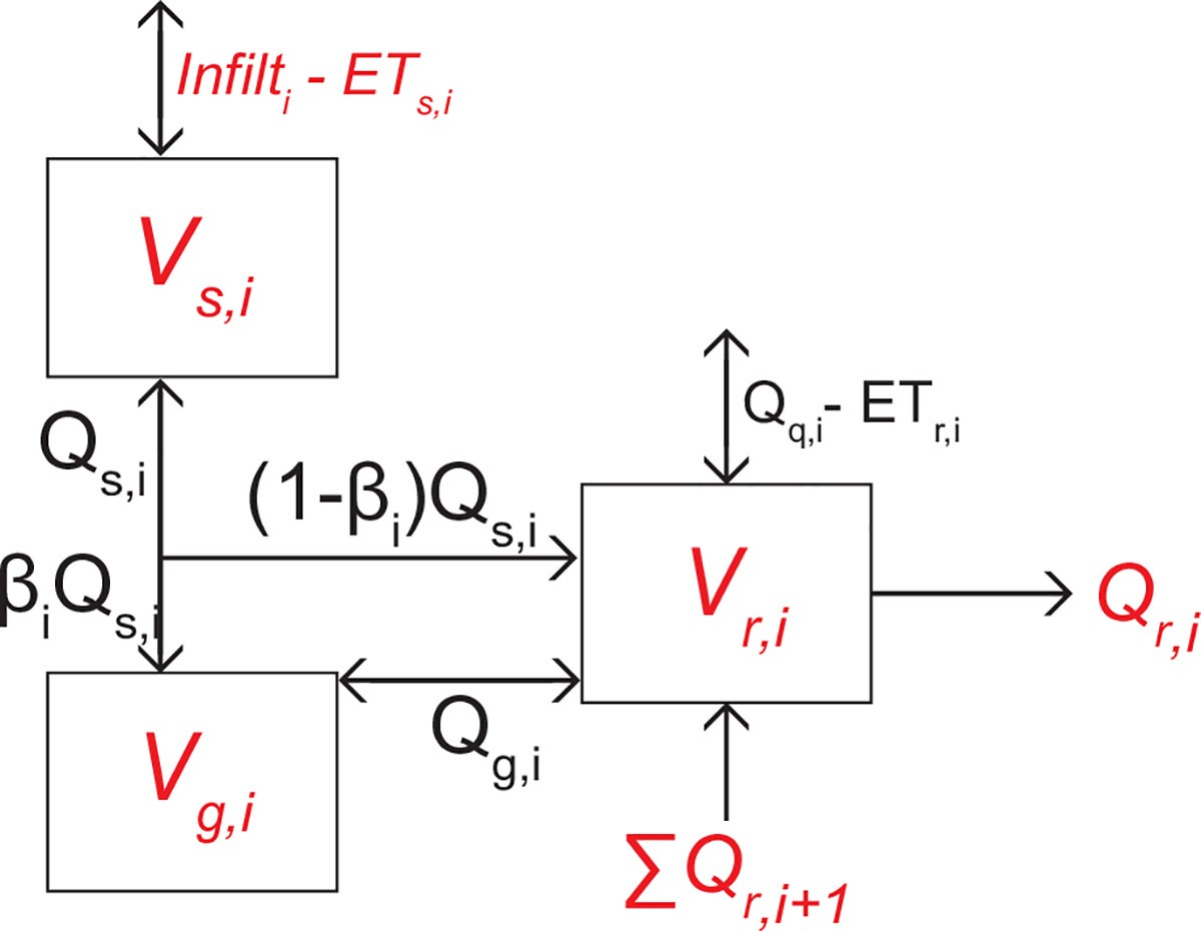
Hydrological box model used in HAN-SoMo. Fluxes and volumes in red italics were extracted from ParFlow-CLM. V_r,i_, V_s,i_, and V_g,i_ are the volumes of water stored in the river, vadose zone and groundwater in sub-watershed i, respectively (m^3^), Q_r,I_, Q_s,i_ and Q_g,i_ are the flows of stream water, vadose zone water, and groundwater, respectively (m^3^ hr^-1^), Infilt_i_ is the infiltration (or exfiltration if negative) (m^3^ hr^-1^), ET_r,i_ is the direct evapotranspiration from surface water (m^3^ hr^-1^), ET_s,i_ is terrestrial evapotranspiration (m^3^ hr^-1^), β_i_ is the base flow index (unitless), and ∑Q_r,i+1_ is the sum of the flow exiting any upstream reaches or tributaries i+1.

We spatially aggregate the ParFlow-CLM simulation outputs to produce a simplified mass balance model based on the conceptual model used by Jackson‐Blake et al. [50]. Watershed hydrology in each sub-watershed *i* is broadly grouped into three pools of water storage: soil water, *V*_*s*,*i*_, groundwater, *V*_*g*,*i*,_ and stream water, *V*_*r*,*i*_ (Fig 2). Soil water represents the total unsaturated (vadose) zone within each sub-watershed, while groundwater represents the saturated zone, to a depth of 30m. Stream water includes all surface water in the main river channel, lakes/ponds, and tributaries. All storages volumes are in m^3^ representing entire sub-watersheds (Fig 1). The key advantage to using ParFlow-CLM to constrain this box model is that it enables us to calculate water residence times for the stream, groundwater and vadose zone that vary with each timestep, rather than assuming they are constant. Through this approach we are further able to account for groundwater recharge and discharge to the stream, as well as bidirectional exchange of water between the vadose zone and groundwater.

Using the time series of fluxes and volumes generated via ParFlow-CLM, the remaining fluxes between hydrological pools were back-calculated analytically for each hourly timestep using mass balance equations:

$$V_{s,i}(t+1)-V_{s,i}(t)={\left([Infilt-{ET}_{s}\right]}_{i}-Q_{s,i})\Delta t$$ (1)

where *Q*_*s*,*i*_ is the daily soil water flow exiting (if positive) or entering (if negative) the soil water reservoir (m^3^ hr^-1^), *V*_*s*,*i*_(*t*+1) and *V*_*s*,*i*_(*t*) are the water volumes within the vadose zone at time *t*+1 and *t*, respectively (m^3^), [*Infilt*−*ET*_*s*_]_*i*_ is the infiltration (or exfiltration) minus the ET from the vadose zone (m^3^ hr^-1^), and Δ*t* is the time step of one hour. When *Q*_*s*,*i*_ is positive, the flow is partitioned between what is delivered directly to the stream (runoff) and what is delivered to groundwater. The proportion of *Q*_*s*,*i*_ delivered to groundwater from soil water is determined by multiplying *Q*_*s*,*i*_ by the base flow index, *β*_*i*_, a unitless values from 0–1 that varies for each sub-watershed *i* for the baseflow period (winter), rising hydrograph (early spring), falling hydrograph (late spring-early summer), and monsoon season (late summer to mid-fall) (constrained in Carroll et al. [37]). When *Q*_*s*,*i*_ is positive, the mass balance for the groundwater pool is:

$$V_{g,i}(t+1)-V_{g,i}(t)=(\beta_{i}Q_{s,i}-Q_{g,i})\Delta t$$ (2)

where *Q*_*g*,*i*_ is the flow of water from groundwater to the stream (if positive), or vice versa (if negative) (m^3^ hr^-1^). If *Q*_*s*,*i*_ is negative, the mass balance for *V*_*g*,*i*_ is:

$$V_{g,i}(t+1)-V_{g,i}(t)=(Q_{s,i}-Q_{g,i})\Delta t$$ (3)

The mass balance for the surface water is solved using:

$$V_{r,i}(t+1)-V_{r,i}(t)=\left((1-\beta_{i})Q_{s,i}+Q_{g,i}+{\left[Q_{q}-{ET}_{r}\right]}_{i}-Q_{r,i}+\sum Q_{r,i+1}\right)\Delta t$$ (4)

where (1−*β*_*i*_)*Q*_*s*,*i*_ is interflow, i.e. the flow directly from the vadose zone to the stream (only relevant if *Q*_*s*,*i*_ is positive), ∑*Q*_*r*,*i*+1_ is the sum of the flow exiting any upstream reaches or tributaries in sub-watershed *i+1*, and *Q*_*r*,*i*_ is the flow leaving the reach. [*Q*_*q*_−*ET*_*r*_]_*i*_ is the overland flow delivered directly to the stream, minus direct ET from surface water (m^3^ hr^-1^).

#### 2.4.3. Mechanistic dissolved N model

**Fig 3:**
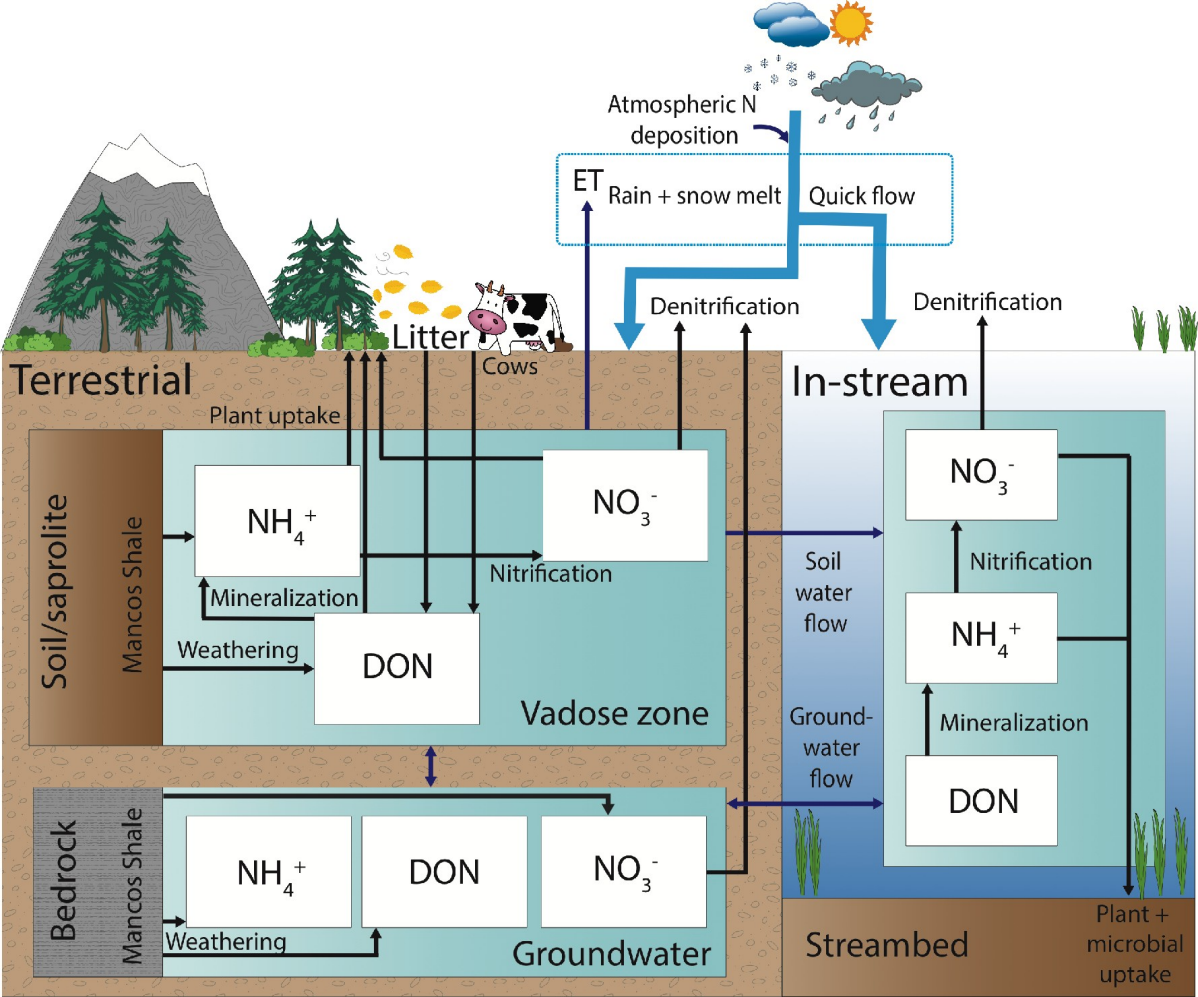
Mechanistic dissolved nitrogen model solved for each sub-watershed in ERW.

NO_3_^-^, NH_4_^+^ and DON pools are modeled for the three hydrologic components (groundwater, soil water, and stream) within each sub-watershed (Fig 3). The majority of fluxes are represented using first order kinetics, with the exception of plant N uptake kinetics, which use Monod kinetics. Note that while the mechanistic N model is also solved using one-hour timesteps, the time units are in days as N reaction parameters are not constrained to resolve diel or hourly trends (e.g. daytime vs. nighttime differences in primary productivity). Hence, all the hydrological inputs described in section 2.4.2 are converted to units of days before insertion into the N model.

In the vadose zone, N pools are solved numerically using:

$$\frac{{dN}_{a,s,i}}{dt}=N_{aP}{\left[Infilt-{ET}_{s}\right]}_{i}-Q_{s,i}N_{a,s,i}+F_{a,dry}A_{i}+0.9[M_{a}\sigma_{i}A_{i}]-F_{a,up,i}-k_{nit,s,i}N_{a,s,i}+{k_{min,s,i}N}_{o,s,i}+F_{a,fix,i}$$ (5)

$$\frac{{dN}_{n,s,i}}{dt}=N_{nP}{\left[Infilt-{ET}_{s}\right]}_{i}-Q_{s,i}N_{n,s,i}+F_{n,dry}A_{i}+0.9[M_{n}\sigma_{i}A_{i})-F_{n,up,i}+k_{nit,s,i}N_{a,s,i}-{k_{den,s,i}N}_{n,s,i}$$ (6)

$$\frac{{dN}_{o,s,i}}{dt}=N_{oP}{\left[Infilt-{ET}_{s}\right]}_{i}-Q_{s,i}N_{o,s,i}+F_{o,dry}A_{i}+0.9[M_{o}\sigma_{i}A_{i}]-F_{o,up,i}-{k_{min,s,i}N}_{o,s,i}+F_{o,lit,i}+F_{o,cows,i}$$ (7)

where $\frac{{dN}_{a,s,i}}{dt},\frac{{dN}_{n,s,i}}{dt}$, and $\frac{{dN}_{o,s,i}}{dt}$ are the rate of change (mol day^-1^) of vadose zone ammonium, *N*_*a*,*s*,*i*_, nitrate, *N*_*n*,*s*,*i*_, and DON, *N*_*o*,*s*,*i*_, in the sub-watershed *i* (mol), over each timestep *dt*. *N*_*aP*_, *N*_*nP*_, and *N*_*oP*_ are the concentrations of NH_4_^+^, NO_3_^-^, and DON, respectively, deposited atmospherically via precipitation (i.e. wet deposition). Wet atmospheric NH_4_^+^ and NO_3_^-^ concentrations in precipitation are taken from the EPA’s CASTNET [51] monitoring location at Gothic (i.e. RMBL, Fig 1), averaged annually over the time period for which data is available (1989–2016), before being converted to mol m^-3^, assuming the concentration of each nutrient in precipitation is constant throughout the year, at 0.011 mol m^-3^ for NO_3_^-^ and 0.0068 mol m^-3^ for NH_4_^+^. Due to a lack of DON concentration measurements from precipitation, we assumed wet deposition of DON to be 25% of the total dissolved N (TDN), i.e. 0.0058 mol m^-3^ [52–54]. This assumption is consistent with Benedict et al. [55], who found that 25% of the annual wet N deposition in Rocky Mountain National Park, Colorado, was DON. We note a lack of significant change in the long-term concentration trends for NH_4_^+^ and NO_3_^-^ from wet deposition, which we use as justification for an arithmetic average. These concentrations are multiplied by [*Infilt*−*ET*_*s*_]_*i*_ in m^3^ day^-1^, yielding a deposition flux in mol day^-1.^

The *Q*_*s*,*i*_*N*_*a*,*s*,*i*_, *Q*_*s*,*i*_*N*_*o*,*s*,*i*_, and *Q*_*s*,*i*_*N*_*n*,*s*,*i*_ terms describe the advective flow of each N species with *Q*_*s*,*i*._ *F*_*a*,*dry*_, *F*_*n*,*dry*_, and *F*_*o*,*dry*_, are the areal dry deposition rates for ammonium, nitrate and DON, respectively (mol m^-2^ day^-1^), and *A*_*i*_ is the surface area of sub-watershed *i* (Table 1). Dry atmospheric NH_4_^+^ and NO_3_^-^ deposition fluxes were gathered from CASTNET’s Gothic monitoring site [51] and averaged annually for 1989–2018 and converted to mol m^-2^ day^-1^, assuming the deposition is constant per unit area per day throughout the year, at 0.28 mol m^-2^ day^-1^ for NO_3_^-^ and 1.29 mol m^-2^ day^-1^ for NH_4_^+^. As with wet deposition, we assume 25% of the total dry N deposition occurs as DON, i.e. 0.52 mol m^-2^ day^-1^. These fluxes are multiplied by the sub-watershed surface areas in m^2^ to yield total deposition fluxes in mol day^-1^.

*M*_*o*_, *M*_*n*_ and *M*_*a*_ are the areal fluxes of DON, nitrate and ammonium mobilized from the Mancos shale to the sub-surface (mol m^-2^ day^-1^), via desorption, ion exchange, dissolution, or rapid mineralization and nitrification specifically associated with the weathered ions [30]. Given the uncertainty related to the relative magnitude of each specific weathering process, as well as the size of the available N stock in the bedrock for the entire watershed, we model the Mancos weathering and associated mineralization and nitrification as a constant combined input into the model, generating input fluxes for all three N species, which are all calibrated. Calibration of shale weathering fluxes is discussed in detail in Section 2.5. We assume 90% of the Mancos weathering flux takes place in the saprolite within the vadose zone, hence the 0.9 coefficient in the weathering term of Eqs 5, 6 and 7, while 10% is weathering and released to the groundwater from fractured bedrock, based on the findings of Wan et al. [30] for a representative hillslope adjacent to PH. We multiply vadose zone weathering rates by *σ*_*i*_, the proportion of each sub-watershed that is underlain by Mancos shale down to 8m below surface (Table 1) [37].

*F*_*a*,*up*,*i*_, *F*_*n*,*up*,*i*_, and *F*_*o*,*up*,*i*_ are the plant uptake fluxes for NH_4_^+^, NO_3_^-^, and DON. The inclusion of direct plant DON uptake reflects the recent acceptance that this uptake mechanism is ecologically relevant, particularly in N-poor systems like the ERW [13, 56]. The generalized N uptake flux for all N species, *F*_*x*,*up*,*i*_, via plants is solved using Monod kinetics:

$$F_{x,up,i}=\frac{F_{x,max,up,i}N_{x,s,i}}{K_{m,x}V_{s,i}+N_{x,s,i}}$$ (8)

where *N*_*x*,*s*,*i*_ is *N*_*n*,*s*,*i*_, *N*_*a*,*s*,*i*_ or *N*_*n*,*s*,*i*_, *F*_*x*,*max*,*up*_ is the maximum uptake rate for each N species in each sub-watershed (mol day^-1^), constrained based on the proportion of four generalized land cover types in each sub-watershed *i*: deciduous and mixed forest (*ρ*_*df*,*i*_), coniferous forest (*ρ*_*cf*,*i*_), meadow (includes grassland, herbaceous wetlands, and dry shrub/scrub) (*ρ*_*ms*,*i*_), and willowy wetland (*ρ*_*ww*,*i*_), using the following:

$$F_{x,max,up,i}={\alpha_{i}A}_{i}[\left(F_{x,max,df}∙\rho_{df,i})+{(F}_{x,max,cf}∙\rho_{cf,i})+{(F}_{x,max,ms}∙\rho_{ms,i})+{(F}_{x,max,ww}∙\rho_{ww,i}\right)]$$ (9)

where land cover proportions are unitless values between 0–1. The land cover proportions are determined using the USGS National Land Cover Database (2016) (Table 1). The sum of *ρ* values do not equal 1 in each sub-watershed as barren and developed areas (e.g. roads) are assumed to have reaction rate constants equal to 0. *F*_*x*,*max*,*df*_, *F*_*x*,*max*,*cf*_, *F*_*x*,*max*,*ms*_ and *F*_*x*,*max*,*ww*_ are maximum uptake fluxes for each N species by each land cover type (mol m^-2^ day^-1^) (S1 Table in S1 File), and *α*_*i*_ is a unitless temperature correction factor:

$$\alpha_{i}={\vartheta_{s}}^{{(T}_{soil,i}-5)/2}$$ (10)

where *ϑ*_*s*_ is a constant equal to 12, and *T*_*soil*,*i*_ is the soil temperature at each timestep, output from ParFlow-CLM. *F*_*x*,*max*,*df*_, *F*_*x*,*max*,*cf*_, *F*_*x*,*max*,*ms*_ and *F*_*x*,*max*,*ww*_ are calculated using N uptake rates per unit surface root surface area (mol N uptake cm^-2^ root area day^-1)^ from Leadley et al. [57] for NH_4_^+^ and NO_3_^-^ and Zhu and Zhuang [56] for DON. We queried the Fine-Root Ecology Database (FRED, Iversen et al. [58]) to gather median belowground biomass per unit area (g m^-2^) for plant and tree species present in each of the land cover regions, and multiplied this with the N uptake rate per unit root area and the mass per unit root area (MSR) of 0.0017 g cm^-2^ [56, 57, 59] (S1 Table in S1 File). *K*_*m*,*x*_ is the half-saturation constant for each N species (mol m^-3^), constrained using values from Zhu and Zhuang [56], Lipson and Näsholm [13] and Leadley et al. [57] (S1 Table in S1 File).

The rate constants for vadose zone net mineralization, *k*_*min*,*s*,*i*_, denitrification, *k*_*den*,*s*,*i*_, and nitrification, *k*_*nit*,*s*,*i*_ (day^-1^) are similarly defined based on the proportion of each land cover type in sub-watershed *i* and rate constants for each land cover type (Table 2):

$$k_{nit,s,i}={\alpha_{i}A}_{i}[{(k}_{nit,df}∙\rho_{df,i})+{(k}_{nit,cf}∙\rho_{cf,i})+{(k}_{nit,ms}∙\rho_{ms,i})+{(k}_{nit,ww}∙\rho_{ww,i})]$$ (11)

$$k_{min,s,i}={\alpha_{i}A}_{i}[{(k}_{min,df}∙\rho_{df,i})+{(k}_{min,cf}∙\rho_{cf,i})+{(k}_{min,ms}∙\rho_{ms,i})+{(k}_{min,ww}∙\rho_{ww,i})]$$ (12)

and

$$k_{den,s,i}={\alpha_{i}A}_{i}[{(k}_{den,df}∙\rho_{df,i})+{(k}_{den,cf}∙\rho_{cf,i})+{(k}_{den,ms}∙\rho_{ms,i})+{(k}_{den,ww}∙\rho_{ww,i})]$$ (13)

**Table 2:** Calibration parameters for scenarios that yield approximately the same quality fit of surface water NO3- time series data. The three values provided in each litterfall calibration refer to 3 day-of-year intervals: day 1–150, day 151–250, and day 251–365 (day 251–366 in year 2016). If only two values are provided (meadows and herbaceous wetlands), they refer to 2 day-of-year intervals, before day 200 and day 200 to the end of the year.

Parameter	Units	Land cover	Calibration 1 (C1)	Calibration 2 (C2)	Calibration 3 (C3)	No Mancos 1 (NM1)	No Mancos 2 (NM2)	No Cows (NC)
			• low instream	• high instream	• high instream	• low instream	• high instream	• high instream
			• high vz	• low vz	• high vz	• high vz	• low vz	• low vz
			• med gw denit	• low gw denit	• high gw denit	• low gw denit	• low gw denit	• low gw denit
			• med Mancos	• med Mancos	• high Mancos	• no Mancos	• no Mancos	• med Mancos
*k*_*nit*,*s*_	day^-1^	Decid+mixed	2.5	0.05	2.5	2.5	0.05	0.05
		Coniferous	2.5	0.05	2.5	2.5	0.05	0.05
		Meadow	2.5	0.05	2.5	2.5	0.05	0.05
		Willowy wet	2.5	0.09	2.5	2.5	0.09	0.09
*k*_*min*,*s*_	day^-1^	Decid+mixed	2.4	0.2	2.4	2.4	0.2	0.2
		Coniferous	2.4	0.2	2.4	2.4	0.2	0.2
		Meadow	1.8	0.15	1.8	1.8	0.15	0.15
		Willowy wet	2.4	0.7	2.4	2.4	0.7	0.7
*k*_*den*,*s*_	day^-1^	Decid+mixed	1.2	0.001	7.5	0.2	5x10^-4^	0.001
		Coniferous	1.2	0.001	7.5	0.2	5x10^-4^	0.001
		Meadow	1.2	0.001	7.5	0.2	5x10^-4^	0.001
		Willowy wet	6	0.001	7.5	1	5x10^-4^	0.001
*F*_*o*,*lit*_	g N m^-2^ day^-1^	Decid+mixed	0.001, 5x10^-6^, 0.002	6x10^-4^, 3x10^-6^, 0.0012	2x10^-4^1x10^-6^, 4x10^-4^	0.001, 5x10^-6^, 0.002	7x10^-4^, 3x10^-6^, 0.007	0.0014, 7x10^-6^, 0.0028
		Coniferous	*υ* = 0.5	*υ* = 0.3	*υ* = 0.1	*υ* = 0.5	*υ* = 0.3	*υ* = 0.7
		Meadow	5x10^-8^, 5x10^-7^	3x10^-8^, 3x10^-7^	1x10^-8^, 1x10^-7^	5x10^-8^, 5x10^-7^	3x10^-8^, 3x10^-7^	7x10^-8^, 7x10^-7^
		Willowy wet	5x10^-4^, 5x10^-6^, 0.002	3x10^-4^, 3x10^-6^, 0.0012	1x10^-4^1x10^-6^, 4x10^-4^	5x10^-4^, 5x10^-6^, 0.002	3x10^-4^, 3x10^-6^, 0.00125	7x10^-4^, 7x10^-6^, 0.0028
*k*_20,*nit*,*r*_	day^-1^	NA	0.3	5.0	5.0	0.3	5.0	5.0
*k*_20,*up*,*r*_	day^-1^	NA	1.0	2.0	1.0	1.0	2.0	2.0
*k*_20,*den*,*r*_	day^-1^	NA	0.4	1.0	1.0	0.4	0.5	1.0
*k*_*den*,*max*,*g*_	day^-1^	NA	0.02	0.001	2.5	0.001	5x10^-4^	0.001
*M*_*o*_	mol m^-2^ day^-1^	NA	7.5 x 10^−6^	7.5 x 10^−6^	2.25 x 10^−5^	0	0	7.5 x 10^−6^
*M*_*n*_	mol m^-2^ day^-1^	NA	7.5 x 10^−6^	7.5 x 10^−6^	2.25 x 10^−5^	0	0	7.5 x 10^−6^
*M*_*a*_	mol m^-2^ day^-1^	NA	2.0 x 10^−7^	2.0 x 10^−7^	6.0 x 10^−7^	0	0	2.0 x 10^−7^

These fluxes represent totals for the entire sub-watershed vadose zone, hence are able to occur concurrently within the model (e.g. as in Wade et al. [10] and Whitehead et al. [60]. Denitrification, for example, which predominantly occurs under saturated, anoxic conditions, can occur in the vadose zone to reflect the fact that there are local areas of ponded water or a perched water table. These parameters are calibrated as described in section 2.5.

*F*_*a*,*fix*,*i*_ is the flux of NH_4_^+^ added to the system via N-fixing plants, assuming 1% of the meadow plant coverage are N fixers [61]. For each timestep, the N fixation flux is chosen as a random number between 10^−8^ and 10^−4^ mol m^-2^ day^-1^ when the soil temperature is above 0°C. The annual areal fixation approximates rates observed in similar mountain meadow environments where *L*. *argenteus* and *L*. *latifolius* are the N fixing plants [62–64], while allowing for daily variability.

*F*_*o*,*lit*,*i*_ is the release of DON from decomposing plant litter (mol day^-1^) and is calibrated so that the annual plant uptake flux is within 10% of the litter plus cow deposition, based on the assumptions of Zhu and Zhuang [56] (see section 2.5). For meadows, aspens, and willows, litter rates are calculated by randomly selecting litter N release rates (mol m^-2^ day^-1^) from uniform distributions, grouped for different periods of time during the year (e.g. high litter in autumn, low in spring) (values are given in Table 2), enabling daily variability as well as seasonal N litter additions that align with the above assumption. Coniferous litter N release, *F*_*o*,*lit*,*spruce*_ (in g N m^-2^ day^-1^) is constrained by fitting equations to model output described by Grant [65] and Mekonnen et al. [66] (using Grant [67])) to develop the following equations:

$$GPP_{spruce}=\frac{1.51}{0.2375+e^{-0.21T_{air}}};\;R^{2}=0.94$$ (14)

and

$$F_{o,\mathrm{lit},\mathrm{spruce}}=\upsilon \;0.0057\;e^{-0.293\;{\mathrm{GPP}}_{\mathrm{spruce}}};\;R^{2}=0.60$$ (15)

where *GPP*_*spruce*_ is the gross primary productivity of spruce (mg C m^-2^ day^-1^), and *υ* is a unitless scaling parameter used in the calibration (Table 2). (Note that the model converts units in g to mol before solving).

The flux of DON delivered to soil water via cattle excretion, *F*_*o*,*cows*,*i*_ (mol day^-1^) is determined according to:

$$F_{o,cows,i}=n_{cows}G_{i}\left(L_{dung}+L_{urine}\right)$$ (16)

where *n*_*cows*_ is the total number of cows in the watershed, equal to 500, *G*_*i*_ is the proportion of the herd present in sub-watershed *i* on timestep *t*, equal to 0 if none are present and 1 if all 500 are present, and *L*_*dung*_ and *L*_*urine*_ are the loads of DON delivered to the soil per cow per day from dung and urine, respectively. *L*_*dung*_ is equal to 8.6 mol cow^-1^ day^-1^ and *L*_*urine*_ is equal to 15 mol cow^-1^ day^-1^ [68]. These values represent the flux of N that enters the soil; i.e. volatilization of NH_3_ to the atmosphere is accounted for. Each year, *G*_*i*_ is set to 1 in the LT region for July 15 –September 8, and for September 9 –October 15, we assume *G*_*i*_ is equal to 0.6 in ME, 0.2 in Copper, and 0.1 in EAQ and Rustlers, based on the approximate annual grazing schedule of the local ranchers. *G*_*i*_ is set to 0 in all other cases.

In the stream, N pools are solved using:

$$\frac{dN_{a,r,i}}{dt}=\sum N_{a,r,i+1}+k_{m,r}N_{o,r,i}-k_{nit,r}N_{a,r,i}-k_{up,r}N_{a,r,i}-\frac{1}{\tau_{r,i}}N_{a,r,i}+N_{aP}{\left[Q_{q}-{ET}_{r}\right]}_{i}+F_{a,int,i}+F_{a,gw,i}$$ (17)

$$\frac{dN_{n,r,i}}{dt}=\sum N_{n,r,i+1}+k_{nit,r}N_{a,r,i}-k_{den,r}N_{n,r,i}-k_{up,r}N_{n,r,i}-\frac{1}{\tau_{r}}N_{n,r,i}+N_{nP}{\left[Q_{q}-ET_{r}\right]}_{i}+F_{n,int,i}+F_{n,gw,i}$$ (18)

$$\frac{dN_{o,r,i}}{dt}=\sum N_{o,r,i+1}-k_{min,r}N_{o,r,i}-\frac{1}{\tau_{r}}N_{o,r,i}+N_{oP}{\left[Q_{q}-ET_{r}\right]}_{i}+F_{o,int,i}+F_{o,gw,i}$$ (19)

where $\frac{dN_{a,r,i}}{dt},\frac{dN_{n,r,i}}{dt}$, and $\frac{dN_{o,r,i}}{dt}$ are the rate of change of total ammonium, *N*_*a*,*r*,*i*_, nitrate, *N*_*n*,*r*,*i*_, and DON, *N*_*o*,*r*,*i*_, in the river in sub-watershed *i* (mol), over each timestep *dt*. ∑*N*_*a*,*r*,*i*+1_, ∑*N*_*n*,*r*,*i*+1_ and ∑*N*_*o*,*r*,*i*+1_ are the sums of ammonium, nitrate, and DON entering from upstream sub-watersheds *i+1* (if applicable) (mol day^-1^), *k*_*m*,*r*_ is the rate constant for in-stream net mineralization (day^-1^), *k*_*nit*,*r*_ is the rate constant for instream nitrification (day^-1^), *k*_*up*,*r*_ is the rate constant for instream N primary productivity (day^-1^), and *k*_*den*,*r*_ is the instream denitrification rate constant (day^-1^). *τ*_*r*,*i*_ is the timestep-specific in-stream water residence time (in days) for each sub-watershed *i*, equal to $\frac{V_{r,i}}{Q_{r,i}}$, which gives the flux (mol day^-1^) of each nutrient exiting the sub-watershed via streamflow when its inverse is multiplied by the in-stream concentration. Wet atmospheric deposition directly to the stream is modeled by multiplying *N*_*aP*_, *N*_*nP*_, or *N*_*oP*_ by [*Q*_*q*_−*ET*_*r*_]_*i*_.

*F*_*x*,*int*,*i*_ is the flux of ammonium (*F*_*a*,*int*,*i*_), nitrate (*F*_*n*,*int*,*i*_), or DON (*F*_*o*,*int*,*i*_) that enter the stream via interflow (mol day^-1^) (relevant if *Q*_*s*,*i*_ is positive), calculated using:

$$F_{x,int,i}=\frac{{(1-\beta }_{i})N_{x,s,i}}{\tau_{s,i}}$$ (20)

where *N*_*x*,*s*,*i*_ is the amount of each N species in the vadose zone at each timestep (mol), *τ*_*s*,*i*_ is the timestep-specific vadose zone water residence time (days), equal to $\frac{V_{s,i}}{Q_{s,i}}$. If groundwater is discharging to the stream, i.e. *Q*_*g*,*i*_ is positive, the flux of each species to the stream, *F*_*x*,*gw*,*i*_, is equal to:

$$F_{x,gw,i}=\frac{N_{x,g,i}}{\tau_{g,i}}$$ (21)

where *N*_*x*,*g*,*i*_ is the amount of each N species in the groundwater (mol), *τ*_*g*,*i*_ is the groundwater residence time for sub-watershed *i* (days), equal to $\frac{V_{g,i}}{Q_{g,i}}$. If the stream is recharging to groundwater, i.e. *Q*_*g*,*i*_ is negative, the N species recharge fluxes are calculated using:

$$F_{x,gw,i}=\frac{V_{r,i}N_{x,r,i}}{Q_{g,i}}$$ (22)

where *N*_*x*,*r*,*i*_ is the amount of each N species in the stream (mol). All instream rate constants are temperature corrected based on water temperature:

$$k_{y,r}={k_{20,y,r}\vartheta_{r}}^{T_{water,i}-20}$$ (23)

where *k*_*y*,*r*_ represents *k*_*nit*,*r*_, *k*_*den*,*r*_, *k*_*min*,*r*_, or *k*_*up*,*r*_ at time *t*, *k*_20,*y*,*r*_ is any of these rate constants at 20°C, *ϑ*_*r*_ is a constant equal to 1.07 [69], and *T*_*water*,*i*_ is the water temperature (°C) at time *t*. *k*_20,*m*,*r*_ is set to 1.5 day^-1^, based on observations of Catalán et al. [70]) and Cheng and Basu [71] that fresh terrestrial organic material mineralizes quickly upon entering the water column (also supported with data in Bertilsson and Stefan [72]). *k*_20,*nit*,*r*_, *k*_20,*up*,*r*_, and *k*_20,*den*,*r*_ are calibration parameters and specific values are discussed and provided in section 2.5 and Table 2.

Groundwater N pools are solved for each timestep using:

$$\frac{{dN}_{a,g,i}}{dt}=F_{a,vz,i}-F_{a,gw,i}+{0.1[M}_{a}A_{i}]$$ (24)

$$\frac{{dN}_{n,g,i}}{dt}=F_{n,vz,i}-F_{n,gw,i}-{k_{den,g,i}N}_{n,g,i}+{0.1[M}_{n}A_{i}]$$ (25)

$$\frac{{dN}_{o,g,i}}{dt}=F_{o,vz,i}-F_{o,gw,i}+{0.1[M}_{o}A_{i}]$$ (26)

where $\frac{{dN}_{a,g,i}}{dt},\;\frac{{dN}_{n,g,i}}{dt}$ and $\frac{{dN}_{o,g,i}}{dt}$ are the rates of change of groundwater ammonium, nitrate and DON storage over time (mol day^-1^). As with Eqs 5–7, the 0.1 coefficient multiplying the Mancos shale term accounts for the assumption that 10% of the weathering takes place in groundwater. *F*_*a*,*vz*,*i*,_ *F*_*n*,*vz*,*i*_ and *F*_*o*,*vz*,*i*_ represent the flow of each N species between groundwater and the vadose zone (mol day^-1^). When *Q*_*s*,*i*_ is positive (i.e. flow is from the vadose zone to groundwater), the generalized expression for each N species, *F*_*x*,*vz*,*i*_, is solved using:

$$F_{x,vz,i}=\frac{\beta_{i}Q_{s,i}N_{x,s,i}}{V_{s,i}}$$ (27)

Whereas if *Q*_*s*,*i*_ is negative, *F*_*x*,*vz*,*i*_ is solved using:

$$F_{x,vz,i}=\frac{Q_{s,i}N_{x,g,i}}{V_{g,i}}$$ (28)

*k*_*den*,*g*,*i*_ the rate constant for denitrification in the groundwater, calculated according to:

$$k_{den,g,i}=k_{den,max,g,i}{\vartheta_{g}}^{{3.8-T}_{gw}}$$ (29)

where *k*_*den*,*max*,*g*,*i*_ is the maximum denitrification reactivity, in this case for the maximum groundwater temperature of 3.8°C, calibrated as discussed in section 2.5, *ϑ*_*g*_ is a constant equal to 0.3, and *T*_*gw*_ is the groundwater temperature (°C) at time *t*. Groundwater denitrification is assumed to happen primarily during periods of rapid groundwater recharge resulting in water table rise and near-surface anoxia, particularly in floodplains. This flux therefore only occurs when *Q*_*s*,*i*_ exceed 1.0 x 10^5^ m^3^ day^-1^ for an entire sub-watershed, limiting groundwater denitrification to the spring snowmelt and high intensity monsoonal rainfall events in the late summer and early fall. Note that groundwater denitrification differs from vadose zone denitrification, which represents localized regions of saturation that exist throughout the growing season, such as in perched water tables.

### 2.5 Model calibration and uncertainty

The mechanistic N model is manually calibrated for Oct. 1, 2014 –Sept. 30, 2016 (731 days) for all 5 sub-watersheds concurrently to yield consistent reaction rate constants across the watershed, using nitrate concentrations measured at the outlets of each sub-watershed. These first two model years were used for calibration as they represent approximately average snowpack years relative to the 1981–2010 mean [73]. Due to exceptionally low in-stream concentrations, we calibrate using the measured concentrations as opposed to fluxes (i.e., concentrations multiplied by the river discharge). This is because the variability in discharge is very large relative to the stream N concentrations. As a result, the fluxes follow nearly identical temporal trends to discharge, and it becomes impossible to resolve differences in N behaviour when using fluxes to calibrate as they are dwarfed by differences in calibrating. The goodness of fit for the calibrations are checked using root mean square error (RMSE) comparisons of modeled vs. measured data (Table 3).

**Table 3:** Median, mean and ranges of nitrate concentrations in the streams for each sub-watershed, and stream water nitrate calibration root mean square errors (RMSE). Units are μM.

	Copper	Rustlers	EAQ	ME	LT
Median	5.1	4.9	1.6	4.7	4.0
Mean	5.6	5.6	2.3	4.8	4.3
Range	0.2–20.4	0.3–15.3	0.1–20.6	0.1–43.6	0.01–58.1
Case 1 (C1)					
RMSE cal	4.2	3.3	5.2	2.4	3.5
Case 2 (C2)					
RMSE cal	3.9	3.1	6.8	2.2	3.6
Case 3 (C3)					
RMSE cal	3.6	3.3	6.0	2.1	3.8
No Mancos Scenario 1 (NM1)					
RMSE cal	4.3	3.6	5.9	3.2	3.5
No Mancos Scenario 2 (NM2)					
RMSE cal	4.3	3.1	7.3	2.5	3.5
No cows scenario (NC)					
RMSE cal	4.0	3.1	6.7	2.5	3.5

**Fig 4:**
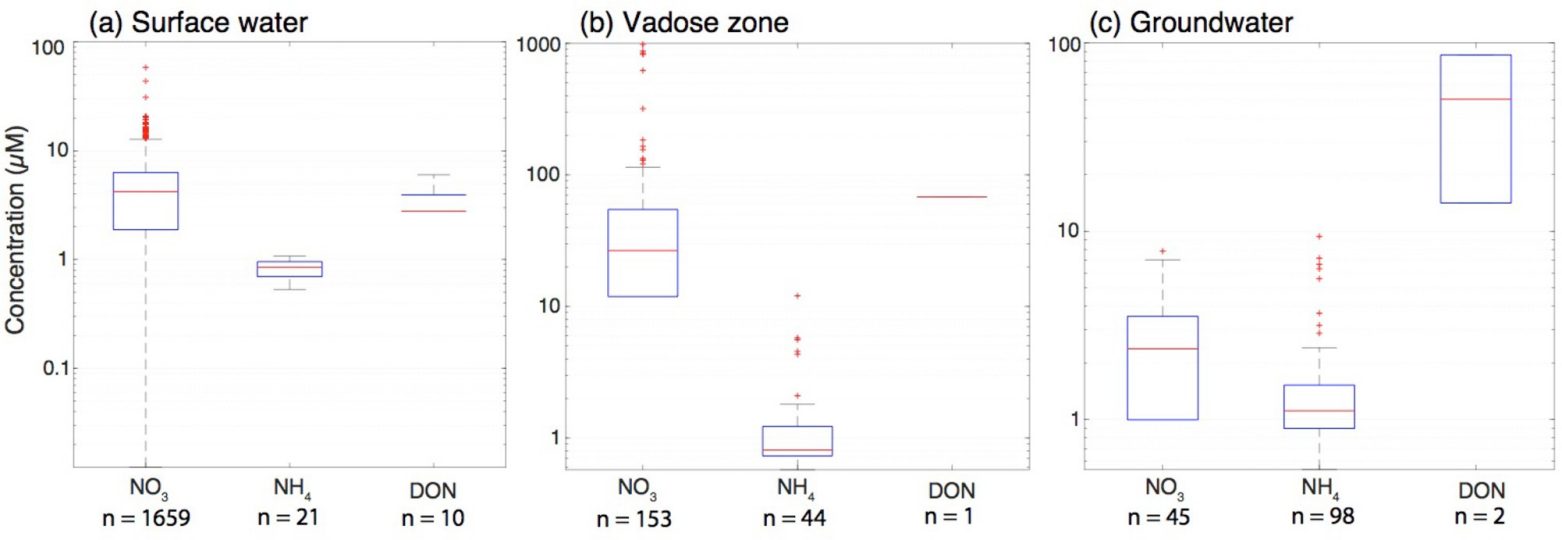
Distributions of nitrogen species concentration measurements in the groundwater, vadose zone and surface water. All vadose zone data is from LT, and >90% of the groundwater data is from LT. Red lines indicate medians, box edges are 1^st^ and 3^rd^ quartiles. For nitrate, ammonium, and dissolved organic nitrogen in surface water, 95% of the concentrations for individual species measured fall below 9.7 μM (0.14 mg N L^-1^), with the outlier concentrations that make up the remaining 5% never exceeding 58 μM (0.81 mg N L^-1^).

Surface water NO_3_^-^ is initialized with concentrations from the chronologically closest measurements. Due to the scarcity of NH_4_^+^ and DON surface water measurements, these pools are initialized based on the median measured concentrations (Fig 4). Given the short surface water residence times, any bias from the initial surface water conditions is eliminated within a few timesteps. Few groundwater and vadose zone measurements for any N species were available throughout the upper watershed due to a limited number of monitoring wells (none in EAQ, Copper and Rustlers) and an absence of vadose zone pore water samplers anywhere except PH. As a result, existing measurements were used to identify the order of magnitude for subsurface concentrations, and the initial conditions were manually adjusted iteratively to ensure that the concentrations during the non-growing season are at or close to steady state (refer to step 5 in calibration procedure below). Groundwater initial conditions are additionally constrained based on the size of baseflow concentrations instream; if initial conditions are set too high, baseflow stream concentrations, which originate overwhelmingly from groundwater discharge, will exceed measured values.

To calibrate the model, we follow this iterative procedure:

1. We assume that atmospheric deposition fluxes, cow N deposition, and plant uptake fluxes are well constrained and cannot be adjusted in the calibration. N-fixation is small and not adjusted.
2. We adjust the magnitude of litter fluxes under the assumption that litter plus cow N release must be within ±10% of the plant uptake flux for the calibration period as done in Zhu and Zhuang [56].
3. The shale weathering flux remains the only source that can be adjusted. We initially use the median value of the total N flux range described in Houlton et al. [5] (9.8 x 10−5–15 x 10^−5^ mol m^-2^ day^-1^) starting with the assumption that DON accounts for half of the total shale weathering flux and that NO_3_^-^ and NH_4_^+^ are the remaining quarters, and adjust each species as necessary. We base these initial ratios on the *in situ* porewater N species concentrations determined at this site by Wan et al. [30] in five boreholes on a hillslope above PH This study showed that weathered N was primarily in the form of DON, followed by NH_4_^+^, but that these species were both quickly mineralized and/or nitrified. Because we do not explicitly model nitrification or mineralization in groundwater, we calibrate N species-specific weathering fluxes to account for these transformations in the subsurface. We assume the shale weathering rate is constant throughout the year and adjust based on the baseflow concentrations in the streams.
4. Denitrification and instream loss are the last sinks to be constrained. Based on our assumption that the total magnitude of N sinks must be within ±10% of the magnitude of sources for the calibration period, these remaining fluxes are adjusted to meet this criterion. We adjust the size of denitrification based on the concentrations in the vadose zone to ensure that the vadose zone NO_3_^-^ pool does not drain or fill. The instream concentrations can then be constrained based on the fluxes that discharge into the river. This process is done iteratively to ensure NH_4_^+^ and DON stream concentrations fall within the approximate range of the measured distributions (Fig 4).
5. We adjust vadose zone and groundwater initial conditions to meet criteria described above. Return to step 1 and rerun until step 5 does not require changes to initial conditions.

This approach leaves several key uncertainties, which we use to explore equifinality. We present three model calibrations representing different regions in the parameter space that fit the surface water NO_3_^-^measurements approximately equally well (Table 2), and discuss drivers of differences in subsurface concentrations, surface water NH_4_^+^ and DON concentrations, and the magnitude of fluxes constrained. To further quantify the relative importance of the Mancos shale as an N source, we develop two additional scenarios that assume the watershed is not underlain by a N-rich shale (Table 2), and investigate whether it is possible to calibrate the model to the measured stream concentrations. Given the high uncertainty already associated with the magnitude of the weathering fluxes, we have not attempted to resolve seasonal differences in the weathering rates as these differences likely already fall within the margin of uncertainty predicted by the various calibration scenarios. Future attempts to resolve the spatially integrated seasonal changes to weathering rates will also need to consider watershed-wide changes to water table height [31, 74]. We also develop a scenario without cattle to quantify the model’s sensitivity to N recycling by cows.

### 2.6 Model scenarios

Through the iterative calibration process described in Section 2.5, we identified six distinct N model cases that represent different regions within the parameter space that fit the data approximately equally well (Table 2). We sought calibration solutions that represent different watershed functionalities, specifically:

- Case 1 (C1): A watershed with prevalent subsurface N cycling, characterized by:
  ○ low instream reactivity, high vadose zone reactivity, moderate Mancos Shale weathering flux, moderate denitrification in groundwater.
- Case 2 (C2): A watershed with prevalent surface water N cycling, characterized by:
  ○ high instream reactivity, low vadose zone reactivity, moderate Mancos Shale weathering flux, low groundwater denitrification.
- Case 3 (C3): A watershed with both surface and subsurface N cycling, characterized by:
  ○ high instream reactivity, high vadose zone reactivity, high Mancos Shale weathering flux, high groundwater denitrification.
- No-Mancos Case 1 (NM1): A watershed functionally equivalent to that of C1, or as close as possible, but without any Mancos Shale weathering
- No-Mancos Case 2 (NM2): A watershed functionally equivalent to that of C2, or as close as possible, but without any Mancos Shale weathering
- No Cows (NC): A watershed functionally equivalent to that of C2, or as close as possible, but without any cattle grazing.

“Low” and “high” refer to relative representative regions within the parameter space where calibrations could be achieved; in other words, while somewhat lower or higher values than what are listed in Table 2 are possible, they exist within a continuum of parameters in the same general section of the parameter space and are thus not identified here because they yield roughly the same conclusions regarding relative importance of N processes. C3 is specifically calibrated to identify the largest shale weathering flux for which a calibration can be achieved.

## 3. Results and discussion

### 3.1 Trends in measured stream water data

**Fig 5:**
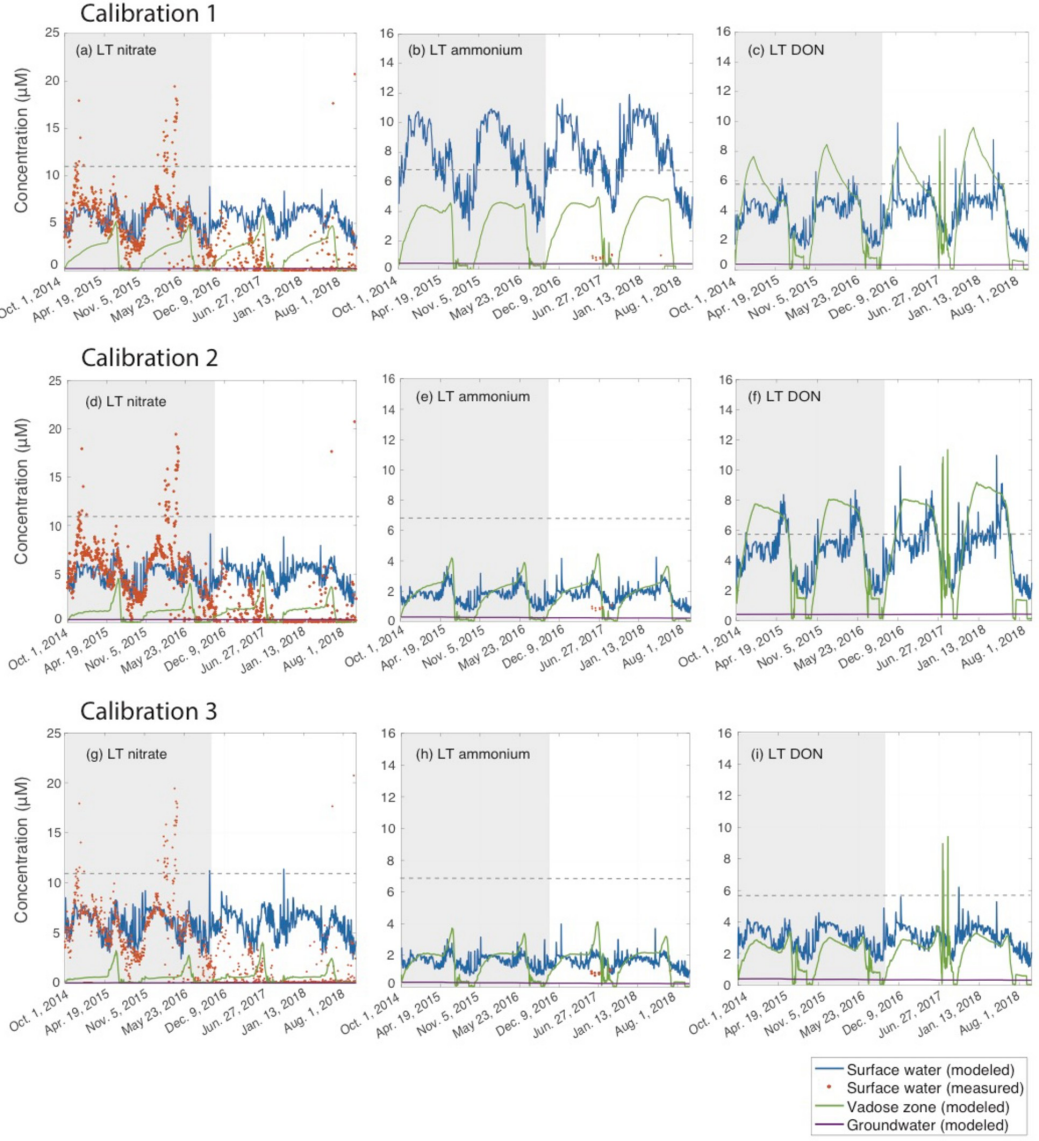
Measured and modeled nitrate, ammonium, and DON concentrations (modeled only) in the stream at outlet of the LT sub-watershed, and the corresponding modeled vadose zone and groundwater concentrations over the four years of model runtime for the three calibrations (C1-C3). Dashed grey lines show the average annual concentration of atmospheric nitrogen recorded at the RMBL CASTNET station, or estimated in the case of DON (refer to section 2.4.3). Grey shading indicates the model calibration period. The time series for the remaining four sub-watersheds can be found in the S1 File, S1–S3 Figs in S1 File, and stream flows in S7 Fig in S1 File.

The model calibration years of Oct. 1, 2014 –Sept. 31, 2016 exhibited approximately average snowpack, relative to the 1981–2010 means, with monthly 2015 January-June snowpack at 98% of average, and 2016 January-June at 123% [73]. Winter 2017 had above average snowpack, with a monthly average at 146% of the mean, while winter 2018 was well below average at only 40% of the mean [73]. During the model calibration period, the two sub-watersheds with the most measured data, ME and LT, show relatively consistent repeating annual trends (Fig 5, S1 Fig in S1 File). Measured NO_3_^-^ concentrations are generally drawn down over the course of the growing season, following the end of the snowmelt (typically beginning in March and ending in June) and rebound throughout the winter, with instantaneous peaks in concentration throughout the year. Spring peaks in NO_3_^-^ concentrations at ME and LT likely correspond with the spring snowmelt, although flows were not measured at ME so this cannot be confirmed.

The following two years of data, Oct. 1, 2016 –Oct. 1, 2018, ME and LT exhibit extremely low concentrations barely above instrument detection limits (consistently below 1 *μ*M from August 2017 through January 2018), with no repeating seasonal trends, and cannot be reasonably used to attempt a separate model calibration with this time period (Fig 5, S1 Fig in S1 File). Given that each of these years experiences substantially different extremes in snowpack, but that stream NO_3_^-^ concentrations in both years remain below those of the average snowpack years, we can only speculate on a mechanism driving these low concentrations. It is possible that during the extremely high snowpack in winter 2017, the vadose zone was sufficiently insulated throughout the winter by deep snowpack that microbial activity could continue to such an extent that dissolved N was lost via denitrification [75, 76]. Additionally, the high snowpack would result in a prolonged period of soil saturation during and following the snowmelt period, further promoting enhanced denitrification. Hence, the dissolved N would not be measured exiting the watershed via the river throughout the growing season as the overall watershed stock was converted to N_2_ or N_2_O gases. In the 2017–2018 winter, which had unusually low snowpack, following this hypothesis we would expect low gaseous emissions due to a lack of snowpack insulation and frozen soils observed throughout the watershed. However, Wan et al. [30] found high winter N_2_O emissions from the subsurface on a hillslope above PH during this time, but since their study did not begin until May 2017, a comparison with the winter before is not possible. Further research is therefore needed to elucidate the driver of consistently low stream NO_3_^-^ concentrations in both the high snowpack and low snowpack years. We discuss the model’s performance in these two years in section 3.6.

### 3.2 Scenario comparison

Across all sub-watersheds, and under all calibration scenarios, stream water NO_3_^-^, NH_4_^+^, and DON concentrations peak during the snowmelt period from March-May, decline during the growing season from May-September, and rebound throughout the fall (Fig 5, S1–S3 Figs in S1 File). When comparing all the calibration scenarios, C2 appears closest to representing the observed watershed dynamics. While the calibration RMSEs for streamwater NO_3_^-^ in each of the sub-watersheds are similar for each calibration (Table 3), C1 is not capable of achieving the low stream NH_4_^+^ concentrations at the LT for which there are measurements available (Fig 5). C2 and C3, which use the large instream reaction rate constants for uptake and denitrification (Table 2), do predict these low concentrations. C2 and C3 instream rate constants are consistent with observations from Boyer et al. [77]. Further support for C2 being the likeliest calibration scenario can be drawn from the magnitude of denitrification, and the size of the denitrification rate constants, which are both more realistic for a N-poor mountainous watershed. For example, McMahon et al. [78] constrained a first order bedrock denitrification rate constant of 0.001 day^-1^, the same as that used in C2, for an analogous N-rich Colorado shale. Similarly, C2 predicts denitrification rates for the subsurface of 3.2 x 10^−4^ mol m^-2^ yr^-1^ (0.044 kg N ha^-1^ yr^-1^), while C3 predicts rates of 0.022 mol m^-2^ yr^-1^ (3.1 kg N ha^-1^ yr^-1^). The latter value is much more characteristic of heavily impacted agricultural systems, while measurements from more pristine alpine and/or forested sites rarely exceed rates of 0.1 kg N ha^-1^ yr^-1^ [79–81].

### 3.3 Atmospheric vs. geogenic N sources

**Fig 6:**
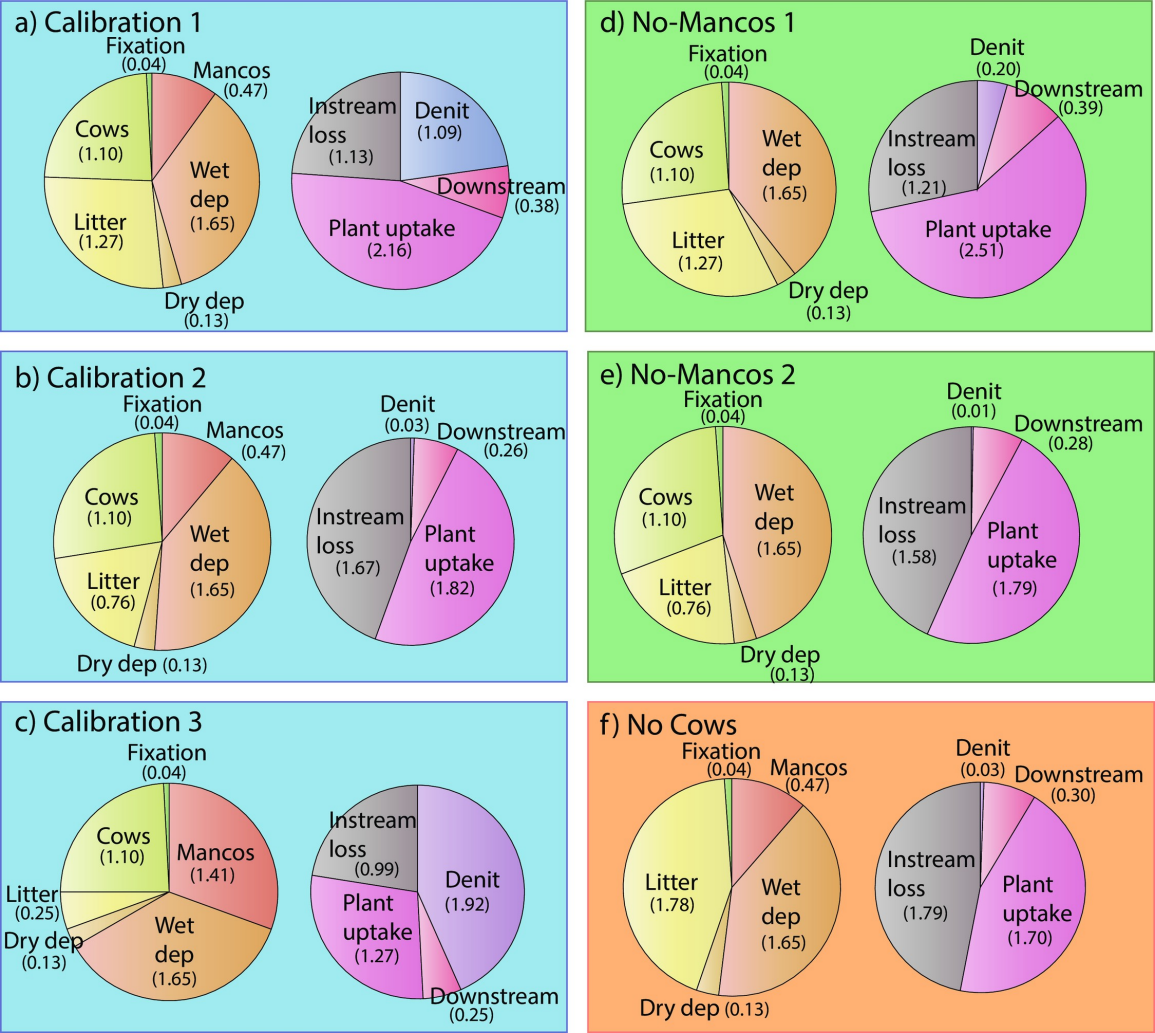
**Proportional breakdown of average annual sources (left pie in each panel) and sinks, or “fates” (right pie in each panel) for the entire ERW for each of the three calibration scenarios (C1-C3), two no-Mancos scenarios (NM1-NM2), and the no cow (NC) scenario.** Average annual fluxes in mol yr^-1^ are given in brackets. Denit = subsurface denitrification. Dry dep = dry deposition. Wet dep = wet deposition. Mancos = Mancos Shale weathering. Instream loss = instream denitrification plus instream biological uptake. Downstream = total N flux sent downstream via the water column at PH.

Over the course of a year, C1-C3 predict that wet atmospheric deposition accounts for an annual average of 1.83 x 10^7^ mol yr^-1^ NO_3_^-^, 1.15 x 10^7^ mol yr^-1^ NH_4_^+^, and 9.84 x 10^6^ mol yr^-1^ DON added to the ERW, or 36–40% of the total sources and 52–74% of the total new sources to the watershed, i.e. the N that is not being liberated from plants or cattle (Fig 6). Dry deposition accounts for 1.73 x 10^4^ mol yr^-1^ NO_3_^-^, 7.99 x 10^4^ mol yr^-1^ NH_4_^+^, and 3.24 x 10^4^ mol yr^-1^ DON, i.e. 1–2% of the sources and 2–3% of the new sources (Fig 6). Comparatively, C1-C3 predict that the Mancos shale weathering supplies 10–31% of the total N mobilized to the ERW’s combined groundwater, vadose zone, and surface water annually (Fig 6), and 21–44% of all new N sources. In the most probable C2 scenario, weathering supplies 12% of the total N mobilized, and 21% of the new N. Annually, DON and NO_3_^-^ weathering fluxes (plus any unaccounted-for subsurface transformations between the species) represent 27–82 mol ha^-1^ yr^-1^ (0.38–1.15 kg N ha^-1^ yr^-1^) each, and NH_4_^+^ fluxes, 7.30 x 10^−5^–2.19 x 10^−4^ mol m^-2^ yr^-1^ (0.010–0.031 kg N ha^-1^ yr^-1^). For comparison, Morford et al. [4] estimated chemical total N weathering rates from N-rich mica-schist in northern California and southern Oregon of 1.6–10.7 kg N ha^-1^ yr^-1^, while Holloway et al. [1] estimated that more than 10 kg N ha^-1^ yr^-1^ originated from biotite schist and diorite saprolite in the Mokelumne River watershed in central California. We note that these are warmer environments that receive more of their precipitation as rain than the ERW, driving higher weathering rates. Our estimates additionally fall below the montane chemical N weathering rates estimated by Houlton et al. [5], which exceed 5 kg N ha^-1^ yr^-1^.

Approximately the same quality calibration can be achieved using the NM1 and NM2 as C1 and C2, respectively (Table 3). These results suggest that geogenic N sources are not the dominant N source to the stream. This finding aligns with that of Holloway and Smith [6], who showed that Mancos shale weathering in the Grand Valley on the western Colorado border did not impact streamwater N concentrations. This conclusion is further supported by a lack of correlation between the amount of shale saprolite within each sub-watershed and instream NO_3_^-^ concentrations. The vadose zone in the EAQ sub-watershed is composed of 70% Mancos saprolite (Table 1), the highest of the sub-watersheds, but has the lowest stream NO_3_^-^ concentrations (Table 3). Model results do predict the highest stream DON and NH_4_^+^ concentrations in EAQ, suggesting that greater coverage of shale saprolite correlates with increased riverine and vadose zone total N. However, the model also predicts very high NO_3_^-^ concentrations in the stream at EAQ, so these trends may reflect the model structural inaccuracies discussed in Section 3.5. Measured low NO_3_^-^ and NH_4_^+^ could arise in EAQ due to the short subsurface residence times that do not provide sufficient time for DON to undergo mineralization and/or nitrification before discharging to the stream.

### 3.4 Terrestrial N-limitation

Litter plus cattle recycling accounting for 46% of the N sources in C2 (with 27% of this value from cattle), and plant uptake accounting for 45% of sinks/fates (Fig 6). Efficient terrestrial plant uptake and recycling via litter deposition, plus low export of dissolved N downstream (<10% of TN sinks, Fig 6) indicates that the ERW is efficiently retaining N. Therefore, these data suggest that the ERW is N-limited with regard to terrestrial plus instream primary productivity [82, 83]. This hypothesis is supported by dissolved inorganic N (DIN = NO_3_^-^ + NH_4_^+^) to DON ratios in the stream that are drawn down to between 0.5–1 in all of the sub-watersheds during the growing season in C2, which has been to shown to be a proxy for N-limited terrestrial systems [84, 85]. This N-retention efficiency suggests that the presence of Mancos shale is not sufficient to liberate plant growth from N-limitation in the watershed. As further evidence of this hypothesis, C2 yields estimates of ~1.0–1.15 x 10^7^ mol for TN stored in the vadose zone and groundwater combined for the entire ERW. Thus, annual Mancos shale weathering fluxes represent 4–5% of the total available N stored in the subsurface. Given that the Parflow-CLM-predicted median groundwater residence times range from 17–50 years in each sub-watershed, nearly all N mobilized to groundwater from geogenic sources remain in groundwater storage for at least a decade before becoming available to the stream or vadose zone for plant uptake or denitrification. Indeed, only ~1.2 x 10^5^ mol NO_3_^-^ is discharged annually from the groundwater to the stream, equivalent to 3–4% of the groundwater NO_3_^-^. In the vadose zone, median residence times range from 20 to 212 days, indicating that geogenic N is more available for plant uptake, denitrification, or export to the stream. While we cannot explicitly track the fate of geogenic N in this type of model, the residence times indicate that, per year, all vadose zone N is entirely replaced at least once, and up to 18 times, with an average of 4.8 x 10^5^ mol TN discharged via interflow to the stream annually.

It is worth emphasizing that in this relatively pristine watershed, the presence of even a small herd of 500 cattle plays a major role in annual N recycling, accounting for a larger flux of DON to the soil than litter in C2, C3 and NM2 (Fig 6). Using the NC scenario, we can equally achieve a calibration without the presence of cows merely by increasing the magnitude of the litter flux. Indeed, mechanistically, roaming cattle may merely serve as an alternative pathway for plant nitrogen to re-enter the soil, with key differences being that cows accelerate the decomposition process and alter the timing of when the N re-entry into the soil takes place. The timing of the cows’ presence in the watershed may greatly impact the extent of their influence on the N cycle. However, given that the grazing period occurs during later summer and early autumn when litter fluxes are highest, their influence on the unperturbed timing of DON release to the soil may be minimal. The key difference between the NC scenario and the equivalent scenario with cows (C2), is that peak vadose zone and stream DON concentrations are ~2–8 *μM* higher in the scenario without cows (Fig 5 and S2 and S6 Figs in S1 File). There are only imperceptible differences in NH_4_^+^ and NO_3_^-^ concentrations. Due to volatilization of ammonia from cow urine and dung (already accounted for in the fluxes used in Eq 16), the overall flux of N into the soil is lower per unit plant biomass than from litter decomposition. In C2, the average annual N sources to the watershed are 3.77 x 10^6^ mol lower than in NC. The continuity of calibrations solutions that can be achieved merely by substituting cow DON release with litter release does however identify the need for DON surface and subsurface water time series and/or ERW-specific cow dung and urine N fluxes to the soil to better constrain this portion of the model.

### 3.5 Surface water N dynamics

We estimate that 2.57–3.97 x 10^5^ mol yr^-1^ TN (0.42–0.65 kg N ha^-1^ yr^-1^) exits the ERW via the river at PH annually (Fig 6). In other words, only 6–9% of the total watershed TN sink exits via the stream in dissolved form. Instream loss via denitrification and primary productivity accounts for 22–46% of the total annual sinks, with C2 representing the upper bound of this range (Fig 6). Indeed, in the C2 calibration, 86% of the N that is delivered to surface water is denitrified or taken up via primary productivity. The magnitude of instream processing is particularly significant, given Parflow-CLM predicts instream water residence times on the order of only days for the ERW, compared with weeks to centuries for the subsurface. As a result, a majority of the N sent downstream may be in the form of particulate organic N (PON). The magnitude of this downstream PON load is likely increased by high physical erosion off steep hillslopes and mountainsides [19, 20]. Fox et al. [86] showed that in the ERW, 23–34% of organic carbon (OC) deposited in the floodplain sediments originated from eroded shale, and speculated that the fraction of N in stream sediments that was derived from shale may been higher. It is therefore likely that our calibrated shale weathering fluxes are underestimates as they do not account for this possible stock within the watershed. Whether or not the magnitude of the unaccounted-for weathering flux results in an overall flux in the most probably C2 calibration scenario that is in excess of the magnitude predicted in C3 remains to be tested.

The downstream flux in the form of PON is particularly important given the number of large dam reservoirs downstream, most notably the Blue Mesa Reservoir on the Gunnison River, approximately 60 km downstream of PH. The Blue Mesa Reservoir has a surface area of 37.15 km^2^ and an annual water residence time of 1.3 years [87], and the TDN concentrations have historically been low (0.1–0.4 mg L^-1^, 7–28 *μ*M), with the majority in the form of DON [88]. Reservoirs, particularly those with long residence times such as the Blue Mesa, are known to eliminate significant N from the water column via burial in sediments or denitrification [89, 90]. A load delivered from upstream in the form of PON would be more readily sedimented than dissolved loads. Indeed, Holloway and Smith [6] estimated that the flux of NO_3_^-^ in the Colorado River at the Colorado-Utah border is 0.049 kg N ha^-1^ yr^-1^, indicating substantial reduction of N fluxes along the river after the ERW. It is therefore worth investigating both the PON fluxes out of the ERW as well as the Blue Mesa Reservoir’s N elimination. The reservoir may act in such a way that “restarts” the upper Colorado River watershed’s N cycle.

The ERW’s N cycles differs from the majority of modeled watersheds in that atmospheric precipitation is nearly always more concentrated in all N species than the porewater and stream water. Thus, during the spring snowmelt, rising instream N concentrations are mainly controlled by flushing of concentrated meltwater through the vadose zone, in addition to direct mixing of meltwater with stream water. This pattern is evident by the abrupt increase in vadose zone and surface water NO_3_^-^, NH_4_^+^ and to some extent DON during the snowmelt period each year (Fig 5). The rising spring concentrations with increased streamflow align with concentration-discharge (C-Q) relationships for N species observed in the adjacent Coal Creek watershed, where positive C-Q trends are shown to be indicative of flushing of more concentrated vadose zone waters in wet seasons [91], while the stream N in dry seasons tends to be sourced from deeper, less concentrated groundwater. The impact that the ERW’s highly concentrated unprocessed atmospheric N has on instream and vadose zone concentrations seems to support the findings of Sebestyen et al. [92], who showed that in northern North American forests, unprocessed atmospheric N can account for a high fraction (defined as > 20% of total N) of these surface water concentrations during wet periods such as snowmelt. While we cannot explicitly track the proportion of vadose zone N that is processed (i.e. its source) once it is inside the box model (Fig 3), the fact that stream and vadose zone N concentrations peak during the largest period of highly concentrated atmospheric N addition suggest that much of the N being delivered to the stream in the spring is unprocessed atmospheric N.

### 3.6 Model uncertainty

Our model development process shows that despite >1600 nutrient concentration and nearly continuous discharge measurements, equifinality is unavoidable for a system of this complexity, where more than 20 parameters are being concurrently calibrated. Indeed, existing biogeochemical modeling research that has managed to overcome equifinality have been limited to only three calibration parameters or fewer [93]. Thus, in order to ask broad questions about subsurface processes like the relative importance of the Mancos shale on watershed N cycling, watershed nutrient modelers need to be prepared to explore multiple calibration solutions. This process enabled us to identify the boundaries of probable parameter ranges and identify the likeliest scenarios based on the magnitudes of specific N processes. We can also use the current modeling experiments to identify a series of knowledge gaps that, once filled, could revise and narrow the HAN-SoMo parameter constraints and further approximate the “true” magnitude of N processes in the ERW. For example, continuous time series of TDN and NH_4_^+^ concentrations can help constrain the magnitude of both instream concentrations and transformation fluxes, such as nitrification and mineralization, in all sub-watersheds. They could also potentially eliminate C1 as a possibility, as longer timeseries of NH_4_^+^ would confirm our hypothesis of high instream reactivity modeled in C2 and C3. TDN measurements can further inform the possible correlation between geogenic N sources and the flux of DON in the stream. Suspended and sediment particulate N time series could help inform both instream uptake fluxes as well as physical erosion rates, thus better constraining the magnitudes of instream productivity as well as the upper bound of the Mancos shale weathering flux.

The implementation of six parameter cases is further useful because it can identify model uncertainties that are consistent across all scenarios. This can guide future exploration, through both measurement and modeling, of unusual N cycling behavior that typically do not emerge for more frequently modeled agricultural systems, where N dynamics are governed mainly by predictable fertilizer application schedules. For example, each of the modeled scenarios predict seasonal trends that repeat annually and do not resolve anomalous or noisy concentration deviations, such as the large peaks in LT NO_3_^-^ in late winter and early spring of 2016 (Fig 5, S1–S3 Figs in S1 File). Given the extremely low concentrations of all N species in each sub-watershed, the ability to identify and quantify repeating seasonal and annual drivers of change in a near-pristine alpine watershed represents an important advancement in watershed nutrient modeling. However, HAN-SoMo performs poorly when attempting to predict atypical biogeochemical circumstances, which seem to occur under unusually high or low snowpacks. For example, in the latter half of the model run period, LT and ME both experience periods where measured stream nitrate concentrations are below modeled output despite similar seasonal patterns and magnitudes of streamflow (S6 Fig), and N concentrations within the major tributaries that are comparable to previous years. The drivers of the extremely low concentrations fall outside the biogeochemical mechanisms described in this model, though may be related to snowpack insulation (see section 3.1), which in itself represents an important conclusion for alpine N modeling. In particular, these findings suggest that end-of-pipe model calibration, which is the norm in watershed nutrient modeling, may not be suitable at low concentrations characteristic of alpine environments. In other words, lumped, semi-distributed models such as HAN-SoMo cannot resolve local hotspots that likely play a disproportionately large role in determining downstream riverine N fluxes. They can, however, be used to identify the local measurements that would eliminate existing uncertainty.

One important example of a local discrepancy identified by the equifinality analysis is the inability to replicate surface water NO_3_^-^ concentrations as low as the measured values at EAQ in any of the calibration cases. The EAQ sub-watershed has the highest fraction of Mancos shale in the saprolite (70%, Table 1), and thus nutrient export here is high in the model. It is important to consider local differences in lithology as a potential source of this discrepancy. While the majority of the ERW is underlain by the upper member of the Mancos shale, an older, lower stratigraphic Mancos unit outcrops upstream of EAQ. This unit is harder and less fractured, partially due to the presence of igneous dikes and sills that cross-cut the shale [94], and thus weathering fluxes calibrated for the remainder of the watershed do not seem applicable. However, even under the ‘no Mancos’ scenarios (NM1 and NM2), modeled EAQ stream concentrations are too high (S4 and S5 Figs). It is possible the spatial and altitudinal heterogeneity in atmospheric deposition throughout the watershed could be an underlying issue. The CASTNET atmospheric deposition measurements collected at RMBL (Fig 1) may be larger than those at higher elevations or different aspects and slopes. Thus, additional measurements of wet and dry deposition would supplement existing measurements, and help identify the spatial variability of N deposition in mountainous systems.

A number of more intensive field-based measurements could be considered to revise model estimates, particularly of the Mancos shale’s weathering fluxes. In the absence of experimental measurements of the rate of N weathering from the Mancos shale cores, field estimates of denitrification time series around the watershed can help constrain gaseous loss to tighten up the overall N mass balance. Additionally, concentration depth profiles for N species in the vadose zone and groundwater at multiple locations and times can help further refine subsurface model constraints. However, given that this strategy would require a large number of depth profiles to constrain spatial variability, it is likely prohibitively expensive throughout much of the ERW. Along these lines, it is worth investigating the role of dissimilatory nitrate reduction to ammonium (DNRA), which, while typically a minor process, could be an important mechanism in local regions of the watershed given the low N concentrations.

## 4. Conclusions

Through exploration of six hypothesized watershed scenarios (with and without shale weathering), we have shown that the shale contributes perceptible concentrations of N to the East River watershed’s N cycle. We note that due to the number of parameters being calibrated and issues of equifinality, the uncertainty associated with the magnitude of the Mancos weathering flux remains high. At most, the Mancos accounts for 44% of the “new” N delivered to the watershed annually. However, this value is likely lower, given the scenario that predicts it (C3) concurrently predicts denitrification fluxes more representative of heavily N-saturated agricultural systems than of a mostly-pristine mountainous wilderness area. Instead, the most plausible watershed scenario, C2, suggests that 21% of the new N delivered to the ERW annually is from geogenic sources, with the remainder deposited atmospherically, and that the ERW is N-limited with regard to terrestrial plus instream primary productivity. Scenario C2 predicts very high in-stream transformation of dissolved N forms to PON, which we note is particularly relevant given the large water storage reservoirs downstream capable of retaining this material in sediment form, potentially “restarting” the upper Colorado River basin’s N cycle. Our modeling approach has further emphasized the uncertainty associated with extremely nutrient poor alpine environments, which are largely neglected in the field of watershed biogeochemical modeling. Through identification of multiple parameter combinations that fit observed data equally well, we have developed a watershed management tool that can be used to describe “typical” seasonal trends, which in turn can guide discussion of anomalous concentration trends and more precise local experimentation and data collection.

## Supporting information

S1 File (DOCX)
