# Supplementary material for: Modeling geogenic and atmospheric nitrogen through the East River Watershed, Colorado Rocky Mountains
Source: PLoS One. 2021 Mar 24;16(3):e0247907. doi: 10.1371/journal.pone.0247907 (PMC7990236; doi:10.1371/journal.pone.0247907)
Supplement: S1 File — (DOCX) [file pone.0247907.s001.docx]

S1 File for

**Modeling geogenic and atmospheric nitrogen through the East River Watershed, Colorado Rocky Mountains**

Taylor Maavara^1,2*^, Erica R. Siirila-Woodburn^1^, Fadji Maina^1^, Reed M. Maxwell^3^, James Sample^4^, K. Dana Chadwick^1,5^, Rosemary Carroll^6,7^, Michelle E. Newcomer^1^, Wenming Dong^1^, Kenneth H. Williams^1,7^, Carl I. Steefel^1^, Nicholas J. Bouskill^1^

^1^ Earth and Environmental Sciences Area, Lawrence Berkeley National Laboratory, Berkeley, CA, USA

^2^ School of the Environment, Yale University, New Haven, CT, USA

^3^ Civil and Environmental Engineering, Princeton Environmental Institute, Princeton University, Princeton, NJ, USA

^4^ Norwegian Institute for Water Research (NIVA), Grimstad, Norway

^5^ Department of Earth System Science, Stanford University, Stanford, CA, USA

^6^ Desert Research Institute, Reno, NV, USA

^7^ Rocky Mountain Biological Laboratory, Crested Butte, CO, USA

*correspondence: taylor.maavara@yale.edu

**Contents:**

- Table S1: Plant uptake parameter constraints
- Figure S1: Calibration 1 output
- Figure S2: Calibration 2 output
- Figure S3: Calibration 3 output
- Figure S4: No-Mancos 1 output
- Figure S5: No-Mancos 2 output
- Figure S6: No Cows output
- Figure S7: Parflow-CLM modeled river flows

| Table S1: Plant N uptake parameters. | | | | | |  |
| --- | --- | --- | --- | --- | --- | --- |
| Parameter | Unit | Deciduous + mixed | Coniferous | Meadow | Willowy wetland | References |
| $F_{a,max}$ | mol m^-2^ day^-1^ | 0.23 | 0.029 | 0.15 | 0.011 | (Chapin III et al., 1979; Iversen et al., 2017; Leadley et al., 1997; Zhu and Zhuang, 2013) |
| $F_{n,max}$ | mol m^-2^ day^-1^ | 0.46 | 0.058 | 0.30 | 0.022 |  |
| $F_{o,max}$ | mol m^-2^ day^-1^ | 0.033 | 0.0041 | 0.022 | 0.015 |  |
| $K_{m,a}$ | mol m^-3^ | 0.2 | 0.2 | 0.2 | 0.2 | (Leadley et al., 1997) |
| $K_{m,n}$ | mol m^-3^ | 0.2 | 0.2 | 0.2 | 0.2 | (Leadley et al., 1997) |
| $K_{m,o}$ | mol m^-3^ | 0.04 | 0.04 | 0.04 | 0.04 | (Lipson and Näsholm, 2001; Zhu and Zhuang, 2013) |

*
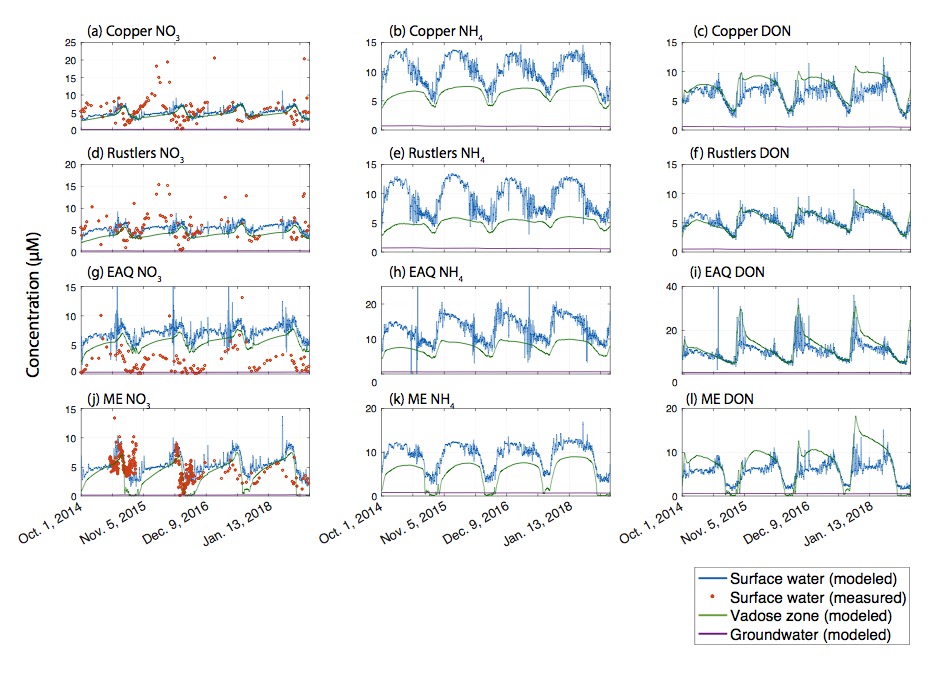
*

*Figure S1: Calibration 1 time series for all nutrient species in upstream sub-watersheds.*

*
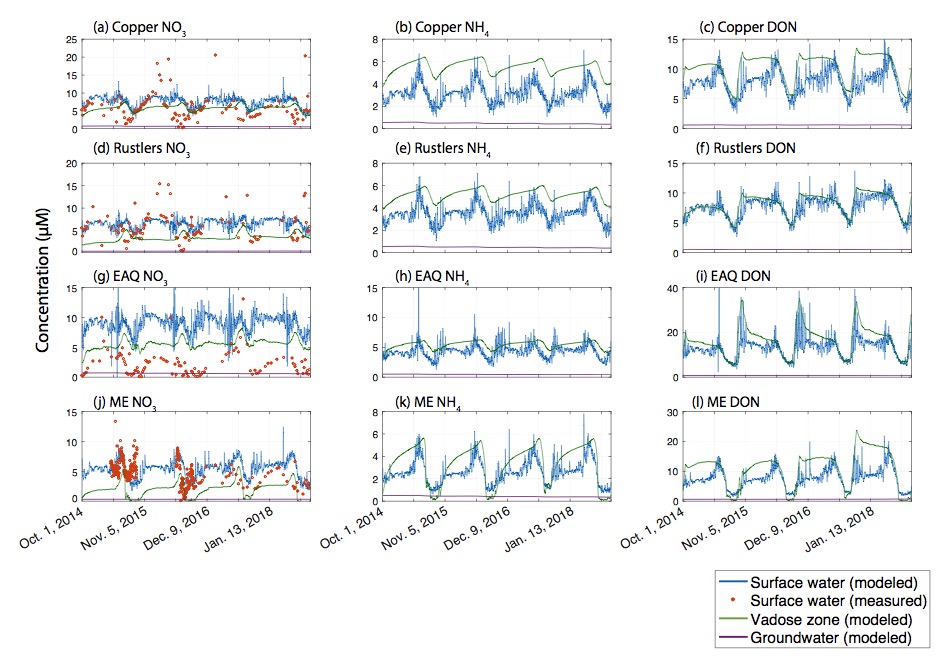
*

*Figure S2: Calibration 2 time series for all nutrient species in upstream sub-watersheds.*


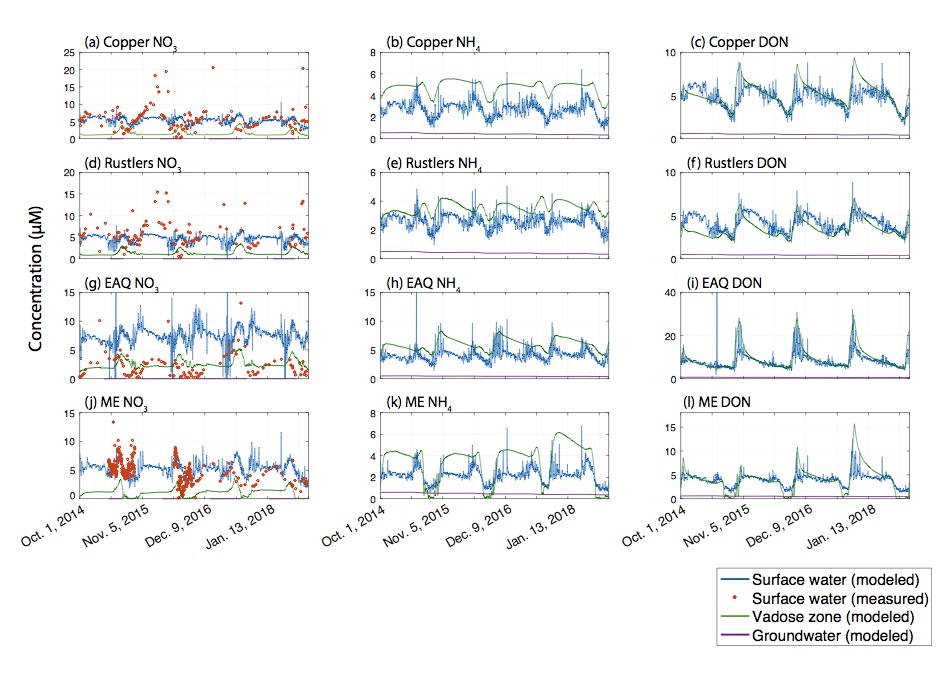


*Figure S3: Calibration 3 time series for all nutrient species in upstream sub-watersheds.*

*
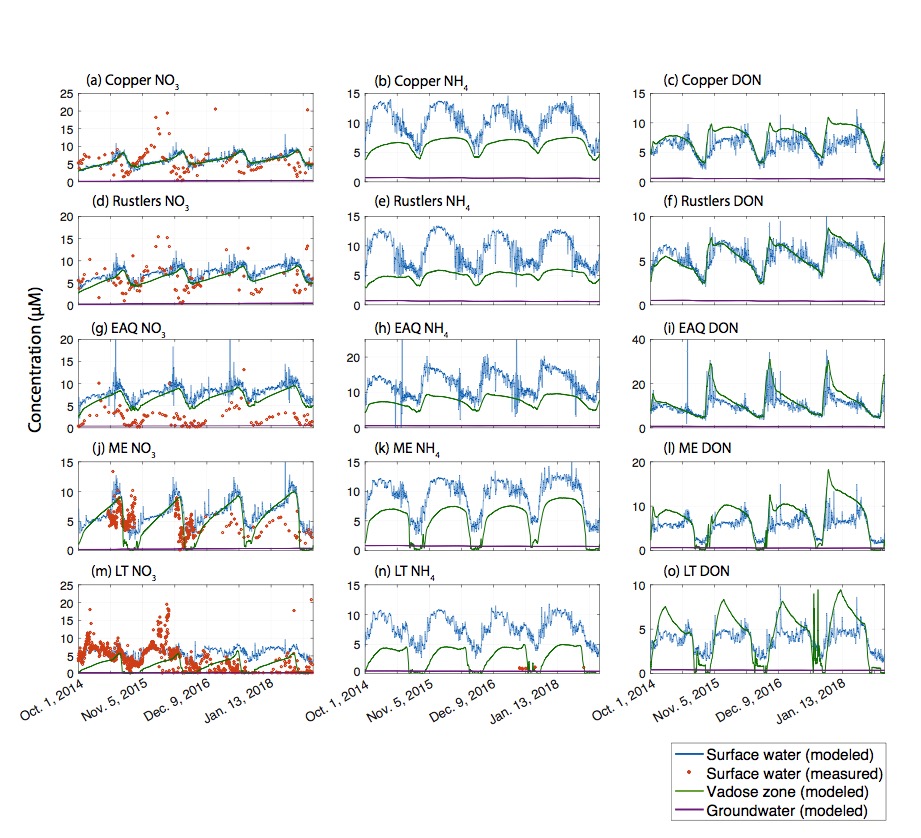
*

*Figure S4: No Mancos Scenario 1 time series for all nutrient species in all sub-watersheds.*

*
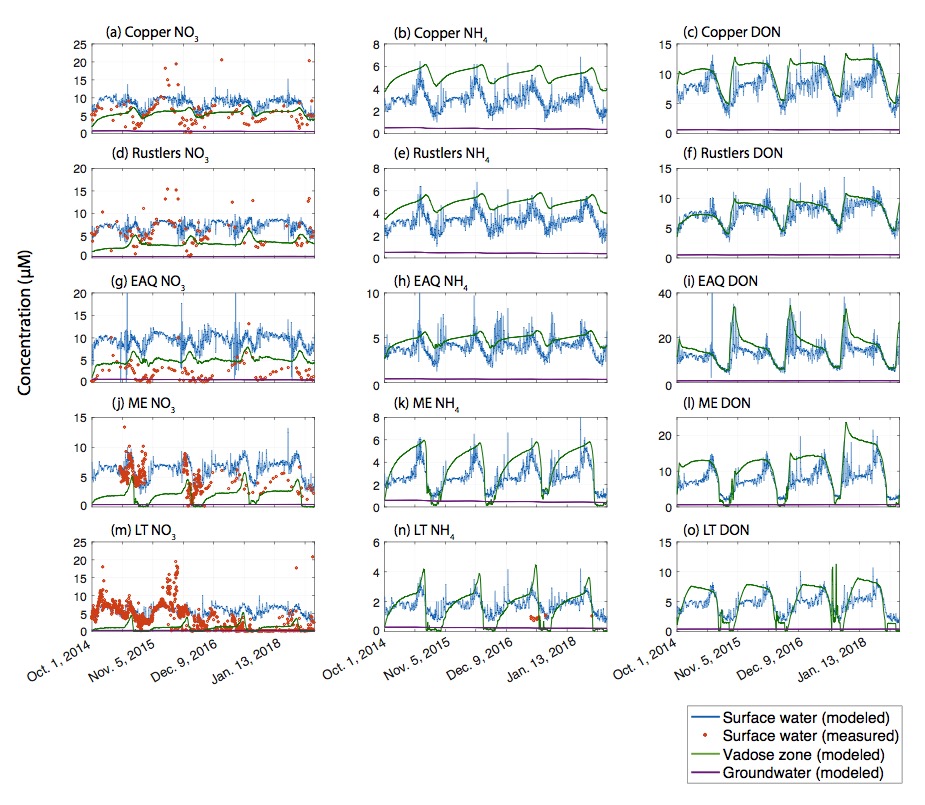
*

*Figure S5: No Mancos Scenario 2 time series for all nutrient species in all sub-watersheds.*

*
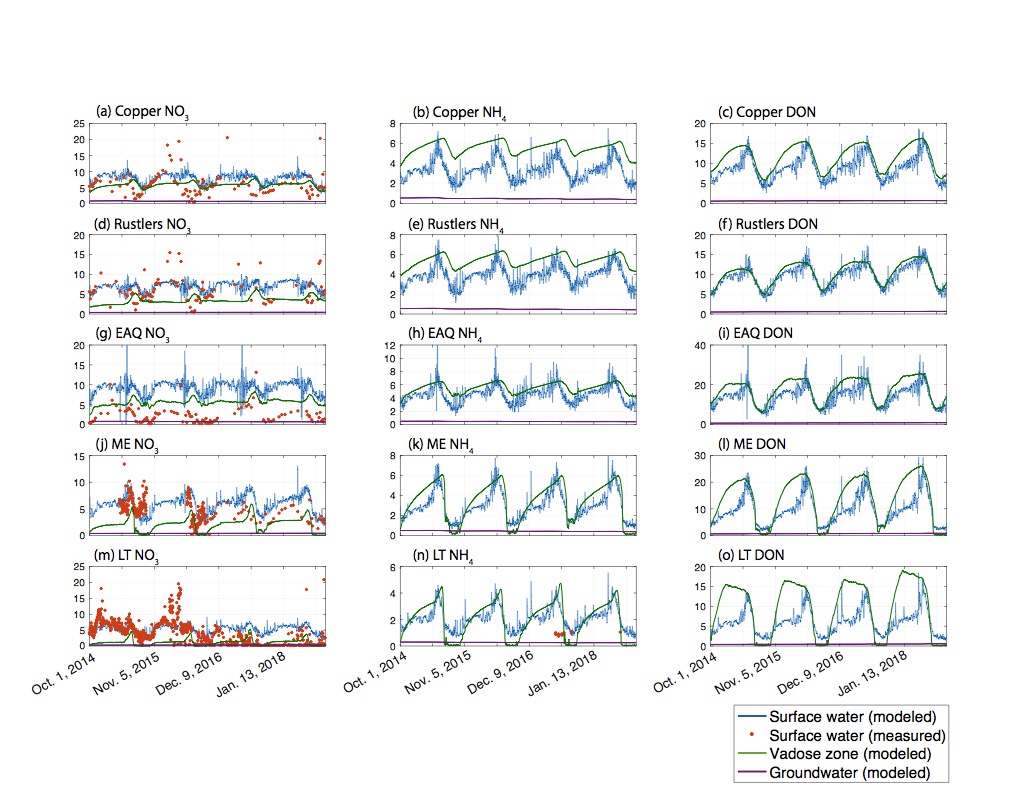
*

*Figure S6: No Cows scenario time series for all nutrient species in all sub-watersheds.*

**

*Figure S7: ParFlow-CLM modeled streamwater discharges for sub-watersheds. For a full discussion of ParFlow-CLM’s model performance in the East River, refer to Foster and Maxwell (2018).*

Chapin III, F.S., van Cleve, K. and Chapin, M.C. (1979) Soil temperature and nutrient cycling in the tussock growth form of Eriophorum vaginatum. The Journal of Ecology, 169-189.

Foster, L.M. and Maxwell, R.M. (2018) Sensitivity analysis of hydraulic conductivity and Manning's n parameters lead to new method to scale effective hydraulic conductivity across model resolutions. Hydrological Processes 33.

Iversen, C.M., McCormack, M.L., Powell, A.S., Blackwood, C.B., Freschet, G.T., Kattge, J., Roumet, C., Stover, D.B., Soudzilovskaia, N.A. and Valverde‐Barrantes, O.J. (2017) A global fine‐root ecology database to address below‐ground challenges in plant ecology. New Phytologist 215, 15-26.

Leadley, P.W., Reynolds, J.F. and Chapin Iii, F. (1997) A model of nitrogen uptake by Eriophorum vaginatum roots in the field: ecological implications. Ecological monographs 67, 1-22.

Lipson, D. and Näsholm, T. (2001) The unexpected versatility of plants: organic nitrogen use and availability in terrestrial ecosystems. Oecologia 128, 305-316.

Zhu, Q. and Zhuang, Q. (2013) Modeling the effects of organic nitrogen uptake by plants on the carbon cycling of boreal forest and tundra ecosystems. Biogeosciences 10, 7943-7955.
